# HDAC6 Inhibition Reduces Seeded Tau and α-Synuclein Pathologies in Primary Neuron Cultures and Wild-Type Mice

**DOI:** 10.1523/JNEUROSCI.1092-25.2025

**Published:** 2025-12-02

**Authors:** Alex Crowe, Yuemang Yao, Mira Newman, Kevt’her Hoxha, Thallyah Baffic, Kurt R. Brunden

**Affiliations:** Center for Neurodegenerative Disease Research, Perelman School of Medicine, University of Pennsylvania, Philadelphia, Pennsylvania 19104

**Keywords:** Alzheimer's, HDAC6, Parkinson's, synuclein, tau, therapeutics

## Abstract

A previous compound screen identified two molecules with histone deacetylase 6 (HDAC6) inhibitory activity that reduced Alzheimer's disease (AD)-like tau inclusions in a primary rat cortical neuron model seeded with AD brain-derived tau fibrils. Testing here of additional HDAC6-selective inhibitors confirmed that compounds of this type decreased neuronal tau inclusions. Moreover, HDAC6 inhibitors also reduced Parkinson's disease (PD)-like α-synuclein aggregates in primary neurons seeded with recombinant α-synuclein fibrils. Knockdown of HDAC6 expression through treatment of seeded neuron cultures with AAV harboring HDAC6-specific shRNA also resulted in a reduction of tau and α-synuclein inclusions. Multiple compounds were evaluated for their ability to inhibit brain HDAC6 in mice, and ACY-738 was found to effectively inhibit brain HDAC6 activity upon oral dosing. ACY-738 was utilized in an efficacy study in which tau and α-synuclein pathologies were induced in wild-type mice through intracerebral injections of AD brain-derived tau and α-synuclein fibrils. Groups of male and female mice first received ACY-738 in drinking water 1 d prior to (preseeding) or 1 week after (postseeding) brain injections of fibrils, followed by continued dosing for an additional 3 months. A control group of fibril-injected mice received water without ACY-738. Immunohistochemical evaluations revealed that ACY-738 administration resulted in significant reductions of tau pathology in both dosing schemes. Moreover, α-synuclein pathology was significantly reduced in mice with preseeding ACY-738 administration, with a strong trend toward reduction after postseeding dosing. These results suggest that HDAC6 inhibitors have potential for the treatment of AD, PD, and related diseases.

## Significance Statement

The spread and abundance of brain tau pathology correlate with AD patient cognitive status, and there are presently no approved drugs that target tau. We demonstrate that HDAC6 inhibition or knockdown reduce both tau and α-synuclein inclusions that develop in wild-type rodent neuron models. A preferred HDAC6 inhibitor, ACY-738, was identified that inhibits brain HDAC6 when administered orally to mice. This compound was examined in a wild-type mouse model that develops concurrent seeded tau and α-synuclein brain inclusions. Significant reductions of both tau and α-synuclein inclusions were observed in mice dosed with ACY-738, suggesting that HDAC6 inhibition may be a therapeutic strategy for AD, PD, and related diseases.

## Introduction

Neuronal somatodendritic inclusions comprised of the tau protein are a defining pathological hallmark of Alzheimer's disease (AD) and related tauopathies (Lee et al., 2001; Kovacs, 2017). The spread and abundance of tau pathology is strongly correlated with AD patient cognitive status (Wilcock and Esiri, 1982; Arriagada et al., 1992), a relationship not observed with the Aβ plaques (Arriagada et al., 1992) that are the other defining AD brain pathology. Recently, monoclonal antibodies that reduce Aβ plaques [e.g., Leqembi (van Dyck et al., 2023) and Kisunla (Sims et al., 2023)] received FDA approval, with evidence of attenuating patient cognitive decline. However, there are no approved drugs that specifically target tau pathology, with tau-directed immunotherapeutics proving ineffective to date in clinical trials (Jabbari and Duff, 2021; Panza et al., 2023). We previously reported results from a small molecule screen that identified modulators of tau inclusions formed within rat cortical neurons after “seeding” with insoluble tau derived from AD brain (AD-tau; Gibbons et al., 2023). Among these tau inclusion inhibitors were the HDAC inhibitors UF010 and BRD73954. The former inhibits multiple HDAC isoforms, including HDAC6, whereas BRD73954 is reported to be a selective inhibitor of HDACs 6 and 8 (Wang et al., 2015).

HDAC6 is the only HDAC family member that localizes to the cytosol (Thomas and D'Mello, 2018; LoPresti, 2020), and prior reports implicate HDAC6 in the regulation of tau protein. HDAC6 inhibition causes HSP90 hyperacetylation and increased degradation of phosphorylated tau in cell lines and primary neurons (Cook et al., 2012). Moreover, HDAC6 inhibition may modulate tau fibril assembly or turnover through a direct effect on tau acetylation (Min et al., 2010; Cohen et al., 2011; Cook et al., 2014; Min et al., 2015). HDAC6 inhibition or knockdown (KD) have also been investigated in mouse models of tauopathy, with conflicting observations. There is evidence of small molecule HDAC6 inhibitors decreasing insoluble brain tau (Choi et al., 2020; Onishi et al., 2021). However, HDAC6 KD in tauopathy mice did not lower tau pathology (Valencia et al., 2021), and HDAC6 knock-out resulted in increased tau pathology and shortened lifespan in tau transgenic mice (Trzeciakiewicz et al., 2020). Thus, it is unclear whether HDAC6 inhibition reduces tau pathology in transgenic mice with tau overexpression.

We describe here the further assessment of HDAC6 inhibitors in models of tau pathology without tau overexpression. Multiple additional HDAC6 inhibitors were shown to cause concentration-dependent reductions of tau inclusions in the rat cortical neuron model. We also examined these HDAC6 inhibitors in a mouse neuron assay that develops Parkinson's disease (PD)-like α-synuclein (αSyn) deposits after seeding with αSyn preformed fibrils (PFFs; Volpicelli-Daley et al., 2014). Notably, the tested HDAC6 inhibitors also decreased neuronal αSyn inclusions. Furthermore, KD of HDAC6 through treatment of neuron cultures with AAV1 expressing HDAC6 shRNA also lowered tau and αSyn inclusions.

The paucity of research on HDAC6 inhibition in PD models and the conflicting literature on HDAC6 regulation of tau pathology led us to assess HDAC6 inhibition in a wild-type (WT) mouse model with concurrent tau and α-synuclein pathology. Initial experiments identified a preferred HDAC6 inhibitor, ACY-738, that reduced brain HDAC6 activity at relatively low doses when administered orally to WT mice. A study was then conducted with ACY-738 in WT mice in which tau and α-synuclein pathologies were induced through intracerebral seeding with AD-tau and αSyn PFFs (Luk et al., 2012b; Guo et al., 2016; Bassil et al., 2021). ACY-738 was orally administered in both preseeding and postseeding dosing schemes for a total of 3 months and the results revealed that the HDAC6 inhibitor led to significant reductions of tau and αSyn pathologies in the preseeding dosing paradigm. Similarly, compound treatment resulted in a significant reduction of tau inclusions in the postseeding dosing scheme, with a strong trend toward reduced αSyn pathology. These results suggest that HDAC6 inhibitors may hold promise for the treatment of AD, PD, and perhaps other neurodegenerative conditions.

## Materials and Methods

### Rat cortical neuron tau inclusion assay and compound testing

Rat cortical neuron cultures were plated on 12-well or 384-well plates. The former were treated with 1 ml/well of 0.1 mg/ml poly-d-lysine (PDL, Sigma-Aldrich) in 50 mM borate buffer, pH 8.5 (Fisher) at 22°C overnight, followed by washing five times with 1 ml of cell culture application water (Lonza). Three hundred eighty-four well plates were purchased precoated with PDL (Corning #356663). Rat cortical neuron preparations were supplied by a core facility (Neurons R Us) at the University of Pennsylvania following protocols approved by the Institutional Animal Care and Use Committee, using previously described methods (Gibbons et al., 2023). Cells were plated at a density of 125,000 cells/well in 12-well plates and 5,000 cells/well in 384-well plates in complete neuronal media supplemented with 5% fetal bovine serum (FBS, Atlanta Biological). Cells were incubated at 37°C with 5% CO_2_ in a humidified incubator (Thermo Fisher Scientific, HERAcell 150i), and after 1 day in vitro (DIV1), media was exchanged to complete neuronal media without FBS such that 12-well plates contained 1 ml/well and 384-well plates contained 50 µl/well. For HDAC6 inhibitor concentration-response analyses, neurons grown on 384-well plates had 25 µl of media removed from each well at DIV7 and 0.1 µl of compound in DMSO or DMSO only was added in triplicate (500× the respective final compound concentration) with a 384-well pin tool dispenser (0.2% final DMSO in all wells). The tested HDAC6 inhibitors were purchased from MedChemExpress. Cells were incubated at 37°C for 1 h, followed by addition of 2.5 ng/well of AD-tau (in 25 µl) that had been sonicated in a water bath sonicator (Diagenode) for 20 cycles (30 s on and 30 s off) at 9°C. The AD-tau was prepared as previously described (Gibbons et al., 2023). Cells were incubated for 15 additional days (DIV22) at 37°C with 5% CO_2_ without exchanging media or adding additional compound. For the 12-well plate immunoblot and tau multimer ELISA assays, compounds were diluted from 10 mM DMSO stock solutions. After removal of 0.5 ml of media, 0.25 ml of compound-containing medium was added (maximum final DMSO concentration of 0.1%). The cultures were incubated at 37°C for 1 h, followed by addition of 0.25 ml of AD-tau that was prepared as above for 384-well assays, with 20 ng/well AD-tau utilized. Cells were incubated for 15 additional days (DIV22) at 37°C with 5% CO_2_ without exchanging media or adding additional compound.

### Mouse hippocampal neuron αSyn inclusion assay and compound testing

Mouse hippocampal neuron cultures on 12-well or 384-well plates were treated similarly to the rat cortical neurons, as described above. Mouse hippocampal neuron preparations were prepared in house following protocols approved by the Institutional Animal Care and Use Committee, using previously described methods (Volpicelli-Daley et al., 2014). Cells were plated at a density of 150,000 cells/well in 12-well plates and 5750 cells/well in 384-well plates in complete neuronal media supplemented with 2% FBS (Atlanta Biological). Cells were incubated at 37°C with 5% CO_2_ in a humidified incubator (Thermo Fisher Scientific, HERAcell 150i), and at DIV1 media was exchanged to complete neuronal media without FBS such that 12-well plates contained 1 ml/well and 384-well plates contained 50 µl/well. For HDAC6 inhibitor concentration-response analyses, neurons grown on 384-well plates had 25 µl of media removed from each well at DIV7 and 0.1 µl of compound in DMSO or DMSO only was added (500× the respective final concentration) in triplicate with a 384-well pin tool dispenser (0.2% final DMSO in all wells). Cells were incubated at 37°C for 1 h, followed by addition of 12 ng/well (in 25 µl) of PFFs made from recombinant S87N human synuclein, prepared as previously described (Luk et al., 2016), that had been sonicated in a water bath sonicator (Diagenode) for 10 cycles (30 s on and 30 s off) at 9°C. S87N-substituted human αSyn fibrils were utilized since they induce robust mouse αSyn pathology (Luk et al., 2016). Cells were incubated for 15 additional days (DIV22) at 37°C with 5% CO_2_ without exchanging media or adding additional compound.

For the 12-well plate immunoblot and synuclein ELISA assays, compounds were diluted from 10 mM DMSO stock solutions. After removal of 0.5 ml of media, 0.25 ml of compound-containing medium was added (maximum final DMSO concentration of 0.1%). The cultures were incubated at 37°C for 1 h, followed by addition of 0.25 ml of S87N human synuclein PFF that was prepared as above for 384-well assays, with 240 ng/well S87N human synuclein PFF utilized due to difference in medium volume between the 12-well and 384-well plates. Cells were incubated for 15 additional days (DIV22) at 37°C with 5% CO_2_ without exchanging media or adding additional compound.

### Immunocytochemical measurement of neuronal tau and αSyn pathology

For immunocytochemical assessment of neuronal tau pathology, rat cortical neurons cultured in 384-well plates that were treated at DIV7 with HDAC6 inhibitors or vehicle only, followed by AD-tau addition, were allowed to incubate until DIV22. At DIV22, wells were washed five times with phosphate-buffered saline, pH 7.4 (PBS) using a plate washer (Bio-Tek ELx405), with 25 µl volume remaining after the final wash. Soluble protein was extracted prior to staining for insoluble rat tau pathology by treating the wells with 25 µl of 2% hexadecyltrimethylammonium bromide (HDTA, Sigma-Aldrich) at 20°C, with HDTA added by a liquid handling system (Perkin-Elmer Evolution P3). After incubating at 22°C for 10 min, the HDTA solution was removed from wells, and 25 µl of 8% paraformaldehyde (PFA, Electron Microscopy Sciences) with 8% sucrose in PBS was added to the wells and incubated at 22°C for 20 min. The extracted wells were washed five times with PBS and treated with blocking buffer [3% bovine serum albumin (BSA, Sigma-Aldrich) and 3% FBS (Corning)] in PBS at 22°C for 1 h. Insoluble pathological rat tau was stained with T49 antibody (Crowe et al., 2020) and DAPI, utilizing a fluorescent-labeled secondary antibody for detection of T49 binding, as previously described (Gibbons et al., 2023).

In most instances, parallel compound-treated 384-well plates were analyzed for MAP2 and DAPI staining to assess neuronal health after treatment with HDAC6 inhibitors. Wells were washed five times with PBS, with 25 µl of PBS remaining after the final wash. Cells were fixed and permeabilized by addition of 25 µl of 8% PFA, 8% sucrose, followed by washing five times with PBS, and then addition of 25 µl of 0.25% Triton X-100 (Sigma-Aldrich) in PBS at 22°C for 20 min. Cells were again washed five times with PBS and then treated with blocking buffer (25 µl) at 22°C for 1 h. Cells were stained with MAP2 antibody (rabbit polyclonal made in house), as well as DAPI, utilizing a fluorescent-labeled secondary antibody for detection of MAP2 staining, essentially as previously described (Gibbons et al., 2023). Images of insoluble tau pathology and neuronal health markers were acquired by high content microscopy on a cell imager (GE HealthCare, IN Cell Analyzer 2200) coupled with a robotic plate handler (PAA, S-Lab), using settings as previously described (Gibbons et al., 2023). Images were collected per well for each staining, using a 10× objective with images analyzed at separate wavelengths for T49-positive tau inclusions and DAPI-stained nuclei. To avoid artifacts on the well edges that result from HDTA extraction, nine separate 1,024 × 1,024 pixel images of T49-positive tau inclusions and DAPI-stained nuclei were taken from the center of the well representing 56% of the total well area. After applying a uniform threshold across the plate to remove background signal at each imaging wavelength, the total area × optical density from the nine separate images were summed to obtain the total signal for the well that was then used for quantification. Fixed and permeabilized wells used for assessment of MAP2 morphology were imaged using the same protocol.

The assessment of αSyn pathology in αSyn PFF-seeded mouse hippocampal cultures treated with HDAC6 inhibitors or vehicle was generally as described above for rat cortical neurons. At DIV22, wells were washed and HDTA-extracted as described above prior to staining for detergent-insoluble αSyn pathology using the 81A antibody (prepared in house, provided by Dr. Kelvin Luk) that recognizes p129S phosphorylated αSyn (Volpicelli-Daley et al., 2014) or the pan-αSyn 9027 antibody (Peng et al., 2018). Parallel compound-treated 384-well plates were analyzed for MAP2 and DAPI staining to assess neuronal health after treatment with HDAC6 inhibitors, as described above. In the study where HDAC6 inhibitors were tested at multiple times after αSyn PFF seeding of neurons, the cultures were fixed and permeabilized as described for MAP2 staining, followed by staining with DAPI and 81A antibody since HDTA extraction is not required to remove soluble αSyn since only insoluble αSyn is stained with the 81A antibody. Plates that were extracted with HDTA prior to staining of total αSyn or phosphorylated αSyn inclusions, as well as DAPI-positive nuclei, had an identical imaging protocol as that above for the assessment of tau pathology. Parallel fixed and permeabilized plates used to visual MAP2 and DAPI staining also used that same imaging protocol. For 81A staining of phosphorylated αSyn and DAPI staining of nuclei on fixed and permeabilized plates, four images of 2,048 × 2,048 pixels were taken representing 100% of the total well area. After applying a uniform threshold across the plate to remove background signal at each imaging wavelength, the total area × optical density from the four separate images were summed to obtain the total signal for the well that was used for quantification. The percentage of tau or αSyn inclusion inhibition by compound treatment was calculated as previously described (Gibbons et al., 2023). Concentration-response curves were adjusted such that the mean at the lowest tested concentration was set as zero for each measure and this baseline adjustment value then applied to all other tested concentrations.

### Immunoblot analysis of soluble and insoluble neuronal tau or αSyn

Rat cortical neuron or mouse hippocampal neuron cultures that were plated in 12-well plates were treated with HDAC6 inhibitors (or vehicle only) followed by addition of AD-tau (cortical neurons) or αSyn PFFs (hippocampal neurons), or vehicle only, at DIV7 as described above. After incubation until DIV22, the cultures underwent media aspiration and wells were washed two times with 1 ml of ice-cold PBS and then cells were lysed in each well using 80 µl of ice-cold RIPA (50 mM Tris, 150 mM NaCl, 5 mM EDTA, 0.5% sodium deoxycholate, 1% NP-40, and 0.1% SDS, pH 8.0) supplemented with a 1:1,000 dilution of protease inhibitor cocktail (prepared in house from Sigma reagents; 1 mg/ml each leupeptin, pepstatin, N*_α_*-tosyl-ʟ-lysine chloromethyl ketone hydrochloride, N-*p*-tosyl-ʟ-phenylalanine chloromethyl ketone, soybean trypsin inhibitor, 100 mM EDTA), a 1:100 dilution of phosphatase inhibitor cocktail (prepared in house from Sigma reagents; 200 mM imidazole, 100 mM sodium fluoride, 100 mM sodium vanadate), and PMSF (Sigma) diluted 1:5,000 from a 0.5 M stock. Each well was scraped with an 11 mm cell scraper (CytoOne) to recover cellular lysates, which were collected and sonicated with 10 pulses using a handheld sonicator (setting 2; Branson XL-2000 series). Lysates from identically treated duplicate wells in the same 12-well plate were pooled together as one sample, with three such pooled samples per treatment to yield triplicate samples. These were subjected to centrifugation at 40,000 × *g* for 30 min. The supernatant fraction was used to assess soluble tau or αSyn species, whereas the pellet was suspended in RIPA at one-fourth of the total supernatant volume for assessment of insoluble tau or αSyn species. The protein concentration in the supernatant fraction was determined by BCA, and supernatant protein (10 µg for rat neuron samples and 10 µg for mouse neuron samples) underwent SDS-PAGE on 10% or 12.5% acrylamide gels of 1.5 mm thickness (for tau or αSyn analyses, respectively), run at 100 volts for 75 min, followed by transfer onto nitrocellulose membranes, as previously described (Yao et al., 2020). Similar gels were run for immunoblotting of HDAC6, with loading of 10 µg of the whole rat neuron extract (prior to separation into supernatant and pellet fractions) and 20 µg of mouse neuron supernatant faction. A volume of the pellet fraction equal to that used to analyze the soluble fraction was also analyzed by SDS-PAGE followed by transfer to nitrocellulose membranes. The amount of rat tau in each fraction was determined by immunostaining the nitrocellulose blots with the T49 antibody (rodent specific tau antibody used at 1:1,000 dilution of ascites), whereas the amount of αSyn in each fraction was assessed with the D37A6 (rodent specific total αSyn; Cell Signaling Technology) and 81A (p129S αSyn; prepared in house, provided by Dr. Kelvin Luk) antibodies. Phospho-tau was assessed with the PHF1 antibody (gift from Dr. Peter Davies, IgG fraction). Secondary antibodies, imaging, and analysis were as previously described (Yao et al., 2020) using a LiCor scanner, with all quantified lanes within the detection limit of the instrument. Quantified tau and αSyn bands from the supernatant fractions were normalized to the corresponding GAPDH integrated value. In the case of insoluble tau and αSyn species, normalization was to the total GAPDH signal from the added supernatant and pellet fractions, with 25% of the pellet fraction GAPDH signal utilized to compensate for differences between the supernatant and pellet fraction volumes.

### Treatment of rat cortical or mouse hippocampal neuron cultures with AAV1 harboring HDAC6 or scrambled shRNA

Rat cortical or mouse hippocampal neuron cultures were grown in 12-well plates as described above. At DIV6, 500 µl of medium was removed and cultures were treated with AAV1 (GeneCopoeia) in 400 µl of medium harboring mouse HDAC6 shRNA (AAV1-A, AAV1-B, or AAV1-C) or nonspecific scrambled shRNA (AAV1-scr) at defined viral particle/ml amounts or vehicle, as specified in the figure legends. AD-tau (rat cortical neurons) or αSyn PFFs (mouse hippocampal neurons) were added on DIV7 in 100 µl to bring the total medium volume up to 1 ml. After incubation until DIV22, the culture wells were washed and lysed as described in a preceding section. For assessment of AAV1-shRNA effects on HDAC6 levels in rat cortical neurons, whole cell lysates were tested for protein content using a BCA assay and equal protein amounts from the different AAV1-shRNA treatments (and vehicle only) were assessed by SDS-PAGE and immunoblotting, using a HDAC6 monoclonal antibody (Proteintech, catalog #16167-1-AP). The HDAC6 signal was quantified using the LiCor system as described in a preceding section, and this value was normalized to the integrated signal of the corresponding lane that had been stained with Ponceau S for normalization to total protein loading. For determination of AAV1-shRNA effect on tau pathology, equal protein amounts from the rat cortical neuron lysate samples underwent testing in the mTau8 multimer ELISA, as described in a later section. For determination of AAV1-shRNA effect on HDAC6 levels in mouse hippocampal neurons, whole cell lysates were separated into soluble and insoluble fractions, as described in a preceding section. The soluble fraction was tested for protein content using a BCA assay, and equal soluble protein fractions from the different AAV1-shRNA treatments (and vehicle only) were assessed by SDS-PAGE and immunoblotting, using the HDAC6 monoclonal antibody. As with the rat cortical neurons samples, the HDAC6 signal was quantified and normalized to the integrated Ponceau S-stained signal from the corresponding lane of the immunoblot. The effect of AAV1-shRNA treatment on insoluble αSyn was determined by analyzing the diluted insoluble protein samples from each treatment with an αSyn ELISA as described below.

### Assessment of neuron and astrocyte morphology after AAV1 treatment

Rat cortical and mouse hippocampal neurons were plated onto glass coverslips coated with PDL as described in a preceding section, with the coverslips placed within 12-well plates. The cultures were grown as described in a preceding section, and at DIV6 AAV1-A, AAV1-B, or no AAV1 was added at 3.12 × 10^7^ viral particles/ml to the mouse hippocampal neurons. Similarly, AAV1-B, AAV1-C, or no AAV1 was added to the rat cortical neurons at 6.25 × 10^7^ viral particles/ml. Either αSyn PFFs (mouse neurons) or AD-tau (rat neurons) was added at DIV7 as described in the preceding sections, and cultures were incubated until DIV22, at which time the coverslips were fixed and permeabilized as described in a preceding section. The coverslips were stained for MAP2 as described above, as well as with GFAP antibody (Agilent/DAKO) to detect astrocytes. Immunofluorescent images were captured at 10× magnification for visual assessment of MAP2-positive dendritic and GFAP-positive astrocyte morphology.

### ELISA analysis of multimeric tau species

Rat cortical neuron culture lysates were prepared as described in an earlier section, without centrifugation to separate soluble and insoluble fractions. Culture lysates were assessed for multimeric tau species using a specific rodent tau-specific antibody, mTau8 (a kind gift from Janssen Pharmaceuticals; Crowe et al., 2020; Gibbons et al., 2023). mTau8 was diluted to 2.5 µg/ml in 100 mM sodium carbonate, pH 9.6 (Sigma-Aldrich) and 30 µl was added per well to MaxiSorp 384-well plates (Thermo Fisher Scientific) followed by centrifugation at 1,000 × *g* for 1 min and storage at 4°C overnight. mTau8-coated plates were washed five times with PBS (prepared from Sigma reagents; 137.9 mM sodium chloride, 2.67 mM potassium chloride, 1.47 mM monobasic potassium phosphate, 8.06 mM dibasic sodium phosphate) plus 0.05% Tween 20 (PBST). Plates were then blocked with heat inactivated blocking buffer consisting of 1% Block Ace powder (Bio-Rad) and 0.05% sodium azide (Sigma-Aldrich) in PBST (90 µl per well). Plates were stored at least 4 d at 4°C prior to further use. Recombinant mouse tau PFFs prepared as previously described (Crowe et al., 2013) were used as standards and were prepared in a twofold dilution series from 320 ng/ml to 20 pg/ml in PBS plus 0.4% BSA. mTau8 was also biotinylated (Thermo Fisher Scientific) for use as detection antibody, with the antibody diluted to 0.125 µg/ml in Buffer C [20 mM sodium phosphate, pH 7.0, 2 mM EDTA, 400 mM sodium chloride, 1% BSA, and 0.005% Thimerosal (Thermo Fisher Scientific)]. Prior to use for ELISA, mTau8-coated plates were washed five times with PBST and patted dry, and then neuronal culture lysates that were diluted 15–60-fold in 0.2% BSA in PBS were added to wells (30 µl total) and centrifuged at 1,000 × *g* for 2 min, and plates were incubated at 4°C overnight. The plates were subsequently washed five times in PBST and patted dry, and 30 µl of biotinylated mTau8 in Buffer C was added to each well followed by centrifugation at 1,000 × *g* for 2 min and incubation at 37°C for 1 h. Plates were again washed five times in PBST and patted dry, followed by addition to each well of 30 µl of streptavidin-HRP (Thermo Fisher Scientific) diluted 1:10,000 in Buffer C followed by centrifugation at 1,000 × *g* for 2 min and incubation at 37°C for 1 h. Plates were washed five times in PBST and patted dry, and 30 µl of 1-Step Ultra TMB (3,3′,5,5′-tetramethylbenzidine, Life Technologies) was added to the wells. After 5–8 min at 22°C to allow colorimetric development, the reaction was quenched by addition of 30 µl of 10% phosphoric acid (Thermo Fisher Scientific), and the absorbance at 450 nm was measured on a SpectraMax iD3 plate reader (Molecular Devices). Sample absorbance which fell within the linear range of the mouse tau PFF standard curve was extrapolated as ng/ml mouse tau, corrected for the dilution factor, and then normalized to the BCA assay protein reading of the lysate to obtain ng rat multimeric tau/mg protein. The values from each treatment group were then normalized to the mean of the vehicle treatment group to get a relative multimeric tau value.

### ELISA analysis of soluble and insoluble αSyn

MaxiSorp 384-well plates (Thermo Fisher Scientific) were coated with 30 µl/well of the pan-synuclein monoclonal antibody 9027 (Peng et al., 2018) at 5 µg/ml in Takeda buffer (100 mM sodium carbonate, pH 9.6) and centrifuged at 1,000 × *g* for 2 min. Plates were incubated overnight at 4°C on a rocking table and were then washed with PBST (prepared as in the preceding section) on a Bio-Tek ELX450 plate washer. After removal of PBST, 80 µl/well of 1% Block Ace (Bio-Rad) in PBS was added, and plates were centrifuged at 1,000 × *g* for 2 min. Plates were then stored at 4°C in Block Ace until needed. Recombinant mouse αSyn standards were prepared in a threefold dilution series from 2,000 ng/ml to 0.4 pg/ml in PBS plus 0.4% BSA. Equal volumes of insoluble mouse neuronal culture homogenate samples from the different treatment conditions were prepared as described in a prior section and were diluted at two dilutions (typically 30- and 60-fold) in PBS plus 0.4% BSA to ensure readings were in the linear absorbance range of the αSyn standard curve. Plates were washed with PBST and patted dry, followed by addition of 30 µl/well of the diluted soluble and insoluble homogenate samples or standards. Plates were centrifuged at 1,000 × *g* for 2 min and then incubated overnight at 4°C. Plates were subsequently washed in PBST and patted dry, followed by addition of 30 µl/well of rabbit anti-mouse synuclein monoclonal antibody (D37A6, Cell Signaling Technology) diluted 1,000-fold in C buffer (20 mM sodium phosphate, pH 7.0, 2 mM EDTA, 1% bovine serum albumin, 400 mM sodium chloride, 0.1% sodium azide). Plates were centrifuged at 1,000 × *g* for 2 min and then incubated at 37°C for 1 h. After incubation, plates were washed in PBST and patted dry, followed by addition of 30 µl/well of biotinylated goat anti-rabbit antibody (Vectastain) diluted 10,000-fold in C buffer. Plates were again centrifuged at 1,000 × *g* for 2 min and incubated at 37°C for 1 h. After another PBST wash, 30 µl/well of avidin-linked HRP diluted 8,000-fold in C buffer was added. Plates were centrifuged at 1,000 × *g* for 2 min and then incubated at 37°C for 1 h, after which plates were allowed to equilibrate to room temperature (∼15 mins). After washing in PBST, 30 µl/well of 1-Step Ultra TMB (3,3′,5,5′-tetramethylbenzidine, Life Technologies) was added, and color was allowed to develop for 5 min, after which the reaction was quenched with 30 µl/well of 10% phosphoric acid (Thermo Fisher Scientific) for 5 min with swirling to allow for full quenching. Absorbance was read at 450 nM on a SpectraMax iD3 plate reader (Molecular Devices). Sample absorbance that fell within the linear range of the αSyn standard curve was extrapolated as ng/ml αSyn and then divided by the protein concentration of the corresponding supernatant fraction (where the vast majority of total protein partitions) to obtain a nominal ng insoluble αSyn/mg protein value. The values from each treatment group were then normalized to the mean of the vehicle treatment group to get a relative insoluble αSyn value.

### Assessment of HDAC6 inhibitor effect on brain AcTub levels

Mouse studies were approved by the University of Pennsylvania Institutional Animal Care and Use Committee (IACUC). Female CD-1 mice (2–3 months of age) received either intraperitoneal or oral gavage doses of vehicle or HDAC6 inhibitors (MedChemExpress) in 30% w/v Kolliphor HS15 (Sigma-Aldrich):9% DMSO:61% PBS at doses indicated in the figure legends. The mice were killed 4 h after dosing, followed by perfusion with 30 ml of PBS. In some studies, mice were given ACY-738 in drinking water at doses indicated in the figure legends. Where indicated, the water contained the following additives per 100 ml (Duan et al., 2004): bovine serum albumin (100 mg), dextrose (3 g), polyethylene glycol 400 (5 ml), and peanut oil (2 ml). The compound dosage was based on mean mouse body weights and the assumption of 5 ml of water consumed per day. Mice that received ACY-738 in drinking water were exposed to compound-containing water for 48 or 72 h, followed by killing and perfusion as above. The combined hippocampus and cortex were dissected from each mouse and placed on ice, followed by homogenization on ice in 0.2 ml of RIPA buffer (50 mM Tris, 150 mM NaCl, 5 mM EDTA, 0.5% sodium deoxycholate, 1% NP-40, and 0.1% SDS, pH 8.0) containing protease inhibitor cocktail (Sigma-Aldrich), 1 mM phenylmethylsulfonyl fluoride (PMSF; Sigma-Aldrich), and 3 µM trichostatin A (Sigma-Aldrich), followed by 20 pulses with a handheld sonicator. The brain homogenates underwent centrifugation at 100,000 × *g* for 30 min at 4°C, and the supernatant was collected. Additional RIPA buffer (0.15 ml) was added to the remaining pellet, which was resuspended and sonicated, with this mixture centrifuged as above. The supernatants from the first and second centrifugation were combined and diluted 10-fold through addition of RIPA, and a BCA assay was performed to determine the protein concentration. The supernatant samples were analyzed for AcTub and α-tubulin levels by ELISA as previously described (Kovalevich et al., 2016). The AcTub to α-tubulin ratio was determined for each mouse, and this value was compared among dosing groups.

### Seeded WT mouse model of concurrent tau and αSyn pathology

All mouse protocols were approved by the University of Pennsylvania IACUC. AD-tau was isolated from AD brain homogenates as previously described (Guo and Lee, 2011; Gibbons et al., 2023), and αSyn PFFs were generated from recombinant full-length mouse αSyn as described (Luk et al., 2012b; Luk et al., 2016). The injection of AD-tau and αSyn PFFs into male and female C57Bl6 mice (2–3 months of age) was essentially as described (Guo et al., 2016; Bassil et al., 2021; Rahayel et al., 2022). Briefly, mice were deeply anesthetized and immobilized in a stereotaxic frame (David Kopf Instruments; Luk et al., 2012b; Guo et al., 2016). All mice received an aseptic unilateral injection of 5 µl (0.4 μl/min using a Hamilton syringe) with a mixture of human AD-tau brain extracts (0.4 µg/μl, 2 μg total) and αSyn PFFs (0.4 μg/μl, 2 μg total) into the right dorsal hippocampus, followed by the overlying cortex as the needle was withdrawn (from bregma, posterior: −2.5 mm; lateral: +2 mm; depth: −2.4 mm and −1.4 mm from the skull). One group of mice (6 males and 6 females) were given *ad libitum* access to drinking water containing ACY-738 (2 mg/kg equivalent dose, assuming 5 ml of water consumption; Selleckchem, catalog #S8648) 1 d prior to injection of AD-tau and αSyn PFFs. A second group of mice (5 males and 6 females) first received ACY-738 in drinking water (2 mg/kg) 1 week after the brain injection of AD-tau/αSyn PFFs. A third group of AD-tau/αSyn PFF-injected mice (5 males and 6 females) received only compound-free water. All mice continued to receive treatment for 3 months after the injection of AD-tau/αSyn PFFs, during which time the mice were monitored for signs of abnormal behavior or distress and were weighed weekly. Upon study completion, the mice were killed and perfused with 30 ml of PBS, followed by removal of the brain and major body organs. The organs were weighed to assess whether there were any compound-induced changes relative to the control mice, and the brains were processed for immunohistochemistry, as previously described (Zhang et al., 2012).

### Measurement of tau and αSyn pathology in the seeded WT mouse model

Paraffin-embedded whole brain sections (6 μm) were prepared from the ACY-738-treated and vehicle (water only) mice. The entire brain of each mouse was sectioned, and every 20 sections underwent immunohistochemical staining for αSyn pathology using the EP1536Y antibody (Abcam) that binds S129 phosphorylated αSyn (Betemps et al., 2023) or for tau inclusions using the AT8 antibody (Thermo Fisher Scientific) that recognizes the phosphorylated S202/T205 residues of pathologic tau (Zhang et al., 2012; Zhang et al., 2018). An initial evaluation of the stained sections revealed that most tau pathology was found between bregma −2.18 and −3.64 and most αSyn pathology between bregma −1.94 and −3.80. Six bregma levels between these regions were evaluated for both the AT8- and EP1536Y-stained sections, with a researcher masked to the treatment group selecting matched bregma sections from each study mouse. The immunostained brain sections were imaged with a 20× microscopic objective using a slide scanner (3DHistech model P250). The ipsilateral hippocampus was manually annotated in all images since this region harbored the preponderance of pathology. The entire hippocampus was outlined at most bregma levels, although the annotation of the more posterior sections excluded a portion of the CA1 region that was devoid of pathology. Importantly, all annotations were done identically at each bregma level by a scientist masked to treatment type. The contralateral hippocampus was also annotated in the AT8-stained sections from bregma −3.64. All images received a uniform thresholding to remove background staining, with quantification of the thresholded AT8- or EP1536Y-positive area and mean optical density performed using HALO (Indica Labs) software, with the evaluator masked to treatment type throughout. The masked immunohistochemical data was then unmasked by another scientist, with all data expressed as the % of annotated area occupied by AT8- or EP1536Y pathology multiplied by the mean optical density of staining (i.e., total integrated pathology as a percentage of hippocampal area). To allow comparison of compound effect across all bregma levels, the mean immunostained value from mice within the vehicle group was used as a divisor for all evaluated sections from all treatment groups at that bregma level, resulting in pathology values that were normalized to the vehicle mean. These normalized pathology values for tau and αSyn at all bregma levels (*n* = 30–36 normalized values/sex/treatment) were then compared by treatment groups. Prior to graphing, all normalized tau and αSyn values for both sexes within a treatment group (*n* = 66–72) underwent outlier analysis using Rosner’s extreme outlier test with a significance value of 0.05 and a maximum of four allowed outliers. There were no identified outliers in the tau AT8 immunohistochemical data, whereas within the αSyn EP1536Y data there were two statistically significant high outliers in the vehicle group, four high outliers in the ACY-738 preventative dosing group, and two high outliers in the ACY-738 interventional dosing group.

### Experimental design and statistical analysis

Compounds were analyzed in triplicate wells at each concentration during concentration-response analyses, and the mean and standard error of the mean are shown in graphs. Concentration-response data were fit to a variable slope sigmoidal curve. For studies with three or more treatment groups, a one-way ANOVA with either a Dunnett's multiple-comparison test relative to the vehicle group, or a Tukey's multiple-comparison test of all groups, was utilized. In one neuronal culture dataset that did not meet a test for normal distribution, a Mann–Whitney test was utilized. These statistics were performed using GraphPad Prism. For neuronal culture studies in which data from more than one neuronal preparation were combined, differences between treatments were assessed using a linear mixed effects model (JASP open-source software) in which treatment type served as the fixed effect and the corresponding values as the dependent variable, with neuron preparation or biological replicates as the random effects variable. Random intercepts were used to account for the correlation between measures, with a Bonferroni’s multiple-comparison correction used for >2 comparisons. A group size of 11 was determined to be appropriate for the WT mouse efficacy study with ACY-738, using a power analysis assuming a power value of 0.8, an alpha value of 0.5, a meaningful difference of 30%, and a 25% standard deviation. Differences between treatment groups in the ACY-738 mouse study were assessed using a linear mixed effects model (JASP open-source statistical software), with the treatment types serving as the fixed effect and the normalized pathology values at each bregma as the dependent variable. Individual mice served as the random effects variable. Random intercepts were used to account for the correlation between measures.

## Results

### HDAC6 inhibitors reduce tau and α-synuclein inclusions in primary neuron culture models

**Figure 1.**
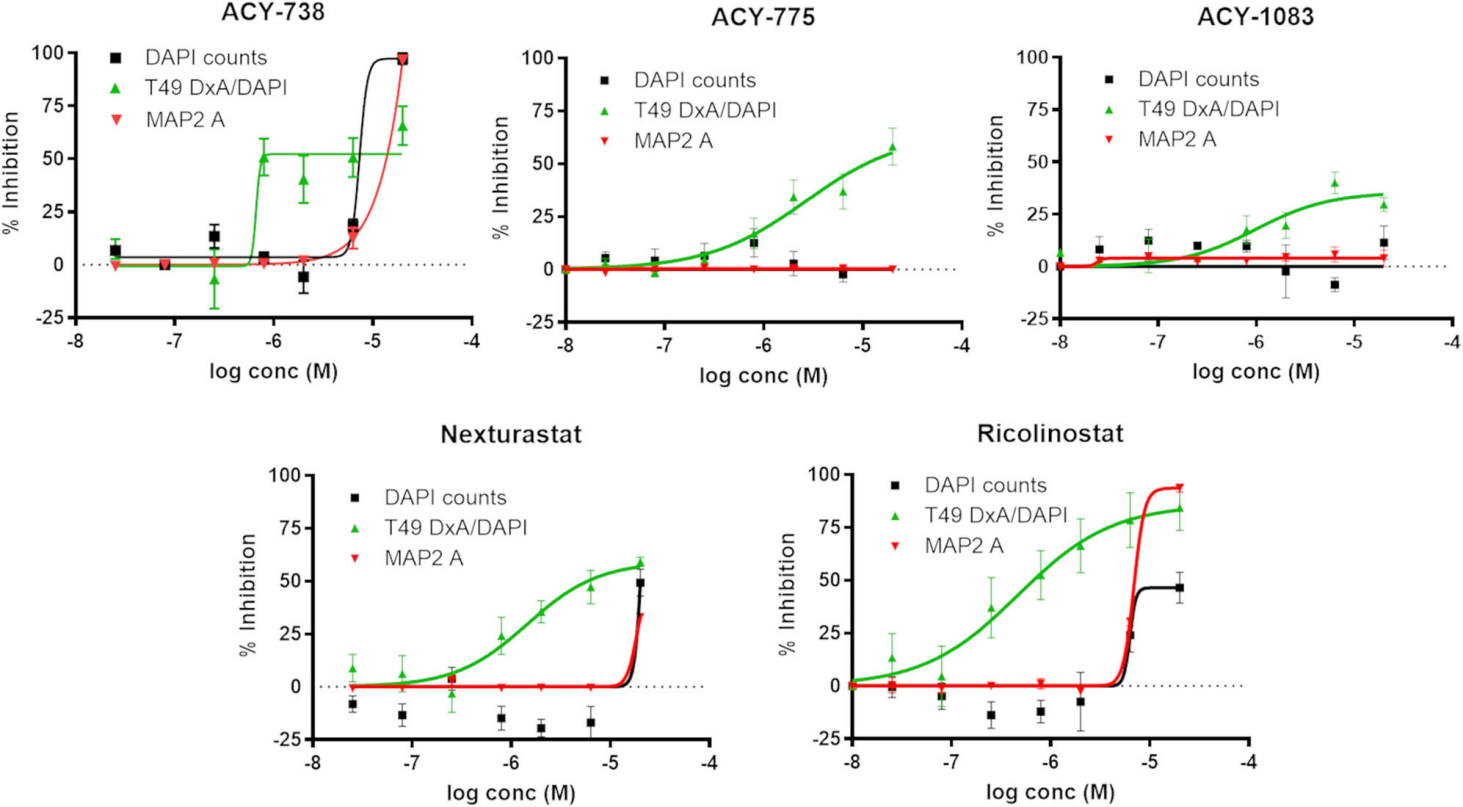
HDAC6-selective compounds reduced intraneuronal tau aggregates. Representative data are shown from replicated studies in which rat cortical neuron cultures were treated at DIV7 with a range of concentrations (10 nM–20 µM) of HDAC6 inhibitors or vehicle in triplicate wells, followed by addition of AD-tau to seed neuronal rat tau aggregates. At DIV22, one set of culture plates underwent HDTA extraction and fixation, followed by DAPI staining of cell nuclei and immunofluorescence staining with T49 antibody to visualize detergent-insoluble rat tau inclusions. Parallel plates that were plated as above were permeabilized and fixed to allow visualization of MAP2-positive neuritic processes. After image analysis, the T49-positive integrated signal [optical density (D) × area (A)] was divided by DAPI counts to get a normalized tau inclusion value, and the percent inhibition relative to vehicle-treated wells was plotted for each compound. Similarly, the percent reductions in DAPI-positive nuclei and MAP2 area relative to vehicle-treated wells were plotted for each compound. Data were fit to a variable slope sigmoidal curve, with error bars representing standard error of the mean.

**Figure 2.**
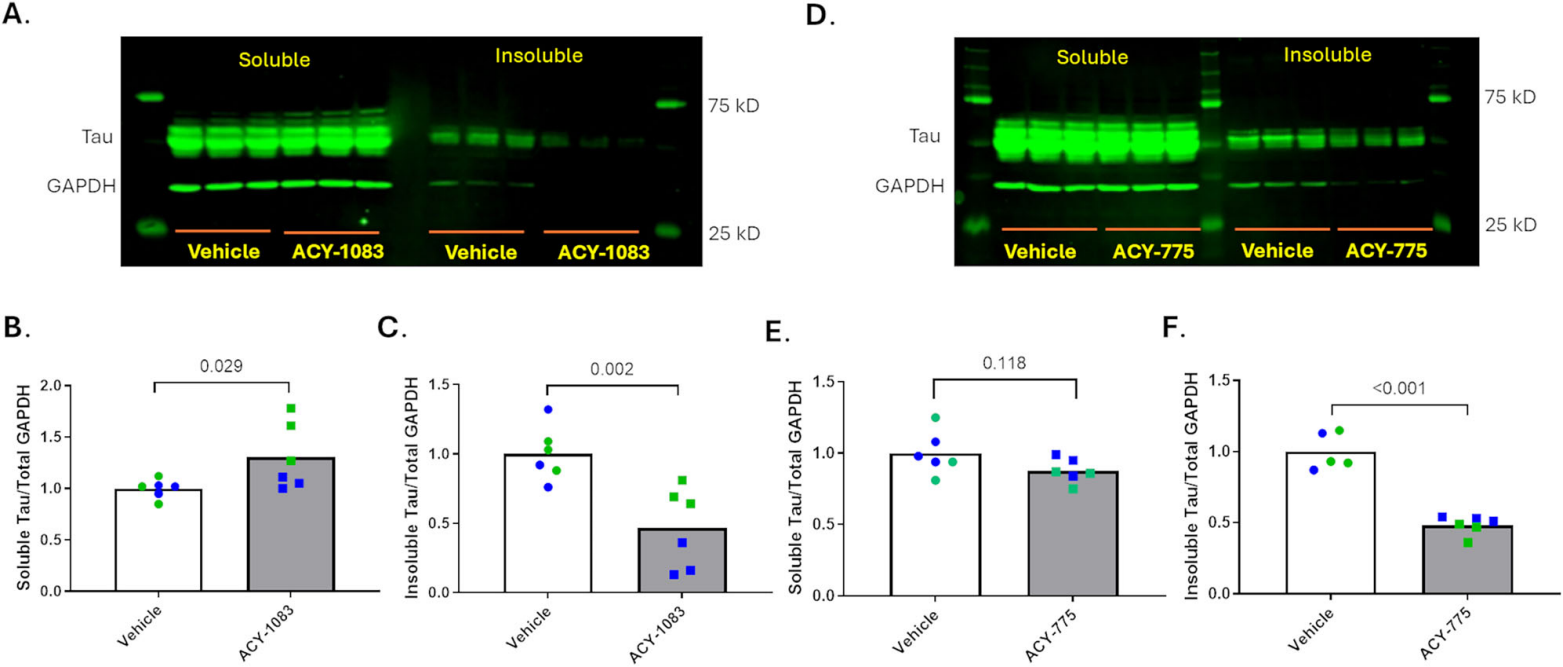
HDAC6 inhibitors reduced insoluble neuronal tau. Two independent sets of rat cortical neuron cultures were treated at DIV7 with vehicle or 6.7 µM of ACY-775 or ACY-1083 followed by AD-tau to seed neuronal tau aggregates. Lysates from identically treated duplicate wells in the same 12-well plate were pooled together as one sample, with three such pooled samples per treatment to yield triplicate samples. At DIV22, the contents of the culture wells were homogenized and fractionated into RIPA-soluble and RIPA-insoluble fractions. Equal total protein amounts of the RIPA-soluble samples were loaded for SDS-PAGE followed by immunoblotting using the T49 antibody to detect rat tau, as well as a GAPDH antibody (representative blots from one dataset are shown in ***A*** and ***D***). The RIPA-insoluble fraction was resuspended in one-fourth the volume of the RIPA-soluble homogenate volume, and a volume of the RIPA-insoluble fraction equal to that used to analyze the RIPA-soluble fraction was loaded for SDS-PAGE followed by immunoblotting as above (***A***, ***D***). The integrated T49 signal for each sample was normalized to the total GAPDH signal (sum of GAPDH in both fractions). The relative change in T49/GAPDH values after compound treatment relative to vehicle treatment were plotted for soluble tau (***B***) and insoluble tau (***C***) after ACY-1083 treatment and soluble tau (***E***) and insoluble tau (***F***) after ACY-775 treatment. Each neuron batch is represented by a different color and each biological replicate by a separate data point, with *p* values calculated using a linear mixed effects model. One high outlier from the vehicle blue data set was omitted from ***F***.

The results of a recent compound screening effort to identify inhibitors of tau inclusion formation in primary rat cortical neurons seeded with AD-tau (Gibbons et al., 2023) revealed two compounds with reported HDAC6 inhibitory activity, UF010 and BRD73954, both of which caused a concentration-dependent inhibition of neuronal tau aggregates. These compounds were not highly specific HDAC inhibitors, although BRD73954 was reported to be a somewhat selective inhibitor of HDACs 6 and 8. To further test whether HDAC6 inhibition results in a reduction of tau pathology, several additional HDAC6-selective inhibitors were examined in the tau inclusion model in which compounds, along with AD-tau, were added to rat cortical neurons cultures at DIV7. As shown in Figure 1 (see also Supplemental Materials, Fig. S1), these HDAC6 inhibitors caused concentration-dependent reductions of neuronal tau aggregates 15 d after compound and AD-tau treatment (DIV22), with activity separated from measures of toxicity (i.e., reduced DAPI-positive nuclei counts or MAP2 dendritic staining). To further confirm that these HDAC6 inhibitors reduced fibrillar tau species, immunoblot analyses were conducted on the soluble and insoluble protein fractions from AD-tau-treated rat cortical neurons incubated with or without ACY-775 or ACY-1083 (at 6.7 µM, a nontoxic high dose). ACY-775 did not cause a significant change in soluble rat tau levels in the AD-tau-treated neurons (Fig. 2*D*,*E*), whereas a small increase of soluble tau (*p* = 0.029) was observed with ACY-1083 (Fig. 2*A*,*B*). Thus, it seems that HDAC6 inhibition has little to no effect on soluble tau in AD-tau-treated neurons. Importantly, both HDAC6 inhibitors caused large and significant 50–60% decreases (*p* = 0.002 for ACY-1083 and *p* < 0.001 for ACY-775) in insoluble rat tau in the AD-tau-seeded cultures (Fig. 2*A*,*C*,*D*,*F*).

**Figure 3.**
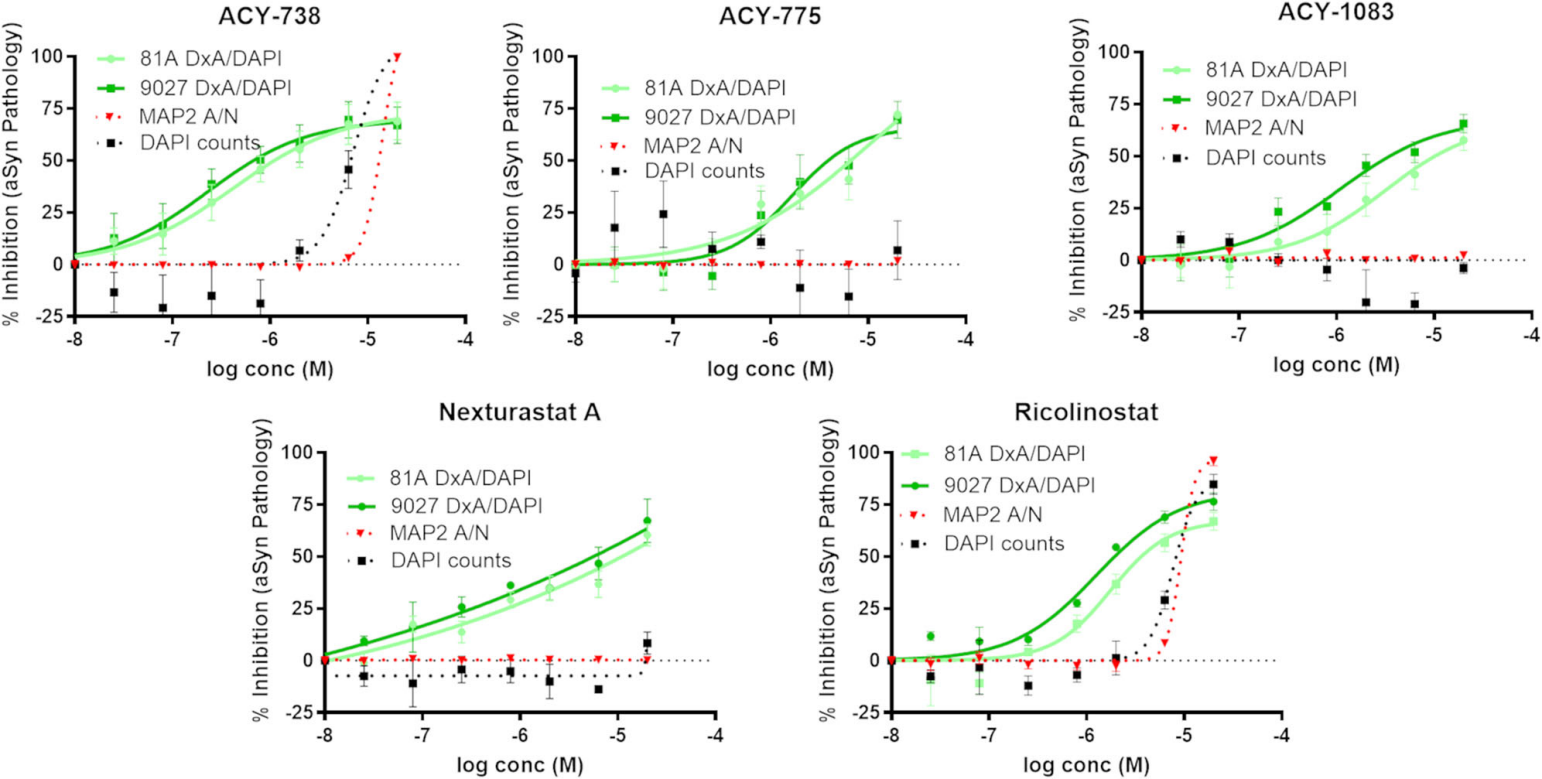
HDAC6 inhibitors reduced intraneuronal αSyn aggregates. Representative data are shown from replicated studies in which mouse hippocampal neuron cultures were treated at DIV7 with a range of concentrations (10 nM–20 µM) of HDAC6 inhibitors in triplicate wells, followed by addition of αSyn PFFs to seed neuronal mouse αSyn aggregates. At DIV22, culture plates underwent HDTA extraction and fixation, followed by DAPI staining of cell nuclei and immunofluorescence staining to allow visualization of 9027-positive αSyn and 81A-positive phospho-S129 αSyn inclusions. Parallel plates that were plated as above were permeabilized and fixed to allow visualization of MAP2-positive neuritic processes. After image analysis, the 9027 and 81A integrated signal values [optical density (D) × area (A)] were divided by DAPI counts to get normalized αSyn pathology values, and the percent inhibition relative to vehicle-treated wells was plotted for each compound. Similarly, the percent reduction in DAPI-positive nuclei and MAP2 area were plotted for each compound. Data were fit to a variable slope sigmoidal curve, with error bars representing standard error of the mean.

**Figure 4.**
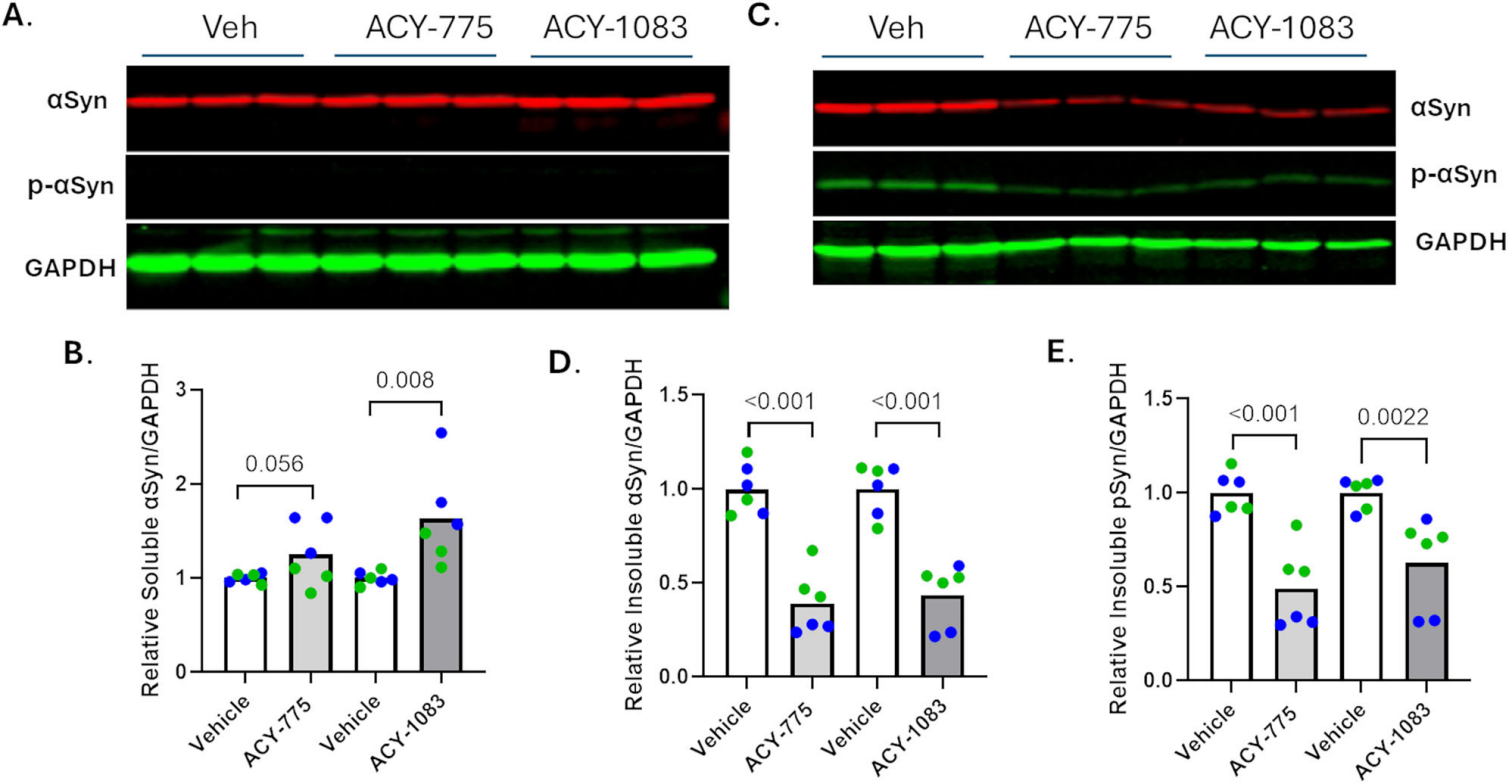
HDAC6 inhibitors reduced insoluble neuronal αSyn. Mouse hippocampal neuron cultures were treated at DIV7 with vehicle or 5 µM of ACY-775 or ACY-1083 followed by αSyn PFFs to seed neuronal mouse αSyn aggregates (two independent neuronal preparations). Lysates from identically treated duplicate wells in the same 12-well plate were pooled together as one sample, with 3 such pooled samples per treatment to yield triplicate samples. At DIV22, the contents of the culture wells were homogenized and fractionated into RIPA-soluble and RIPA-insoluble fractions. Equal protein amounts of the RIPA-soluble samples were loaded for SDS-PAGE followed by immunoblotting using the D37A6 (total mouse αSyn) and 81A (phospho-S129 αSyn) antibodies, as well as a GAPDH antibody, with an immunoblot from one of the neuronal preparations shown in ***A***. The RIPA-insoluble fraction was resuspended in one-fourth the volume of the RIPA-soluble homogenate volume, and a volume of the RIPA-insoluble fraction equal to that used to analyze the RIPA-soluble fraction was loaded for SDS-PAGE followed by immunoblotting as above (***C***). The integrated D37A6 and 81A signals for each sample were normalized to the total GAPDH (sum of that in both fractions). The relative change in values after compound treatment relative to vehicle treatment were plotted for soluble αSyn (***B***), insoluble αSyn (***D***), and insoluble phospho-αSyn (***E***). Each neuron batch is represented by a different color and each biological replicate by a separate data point. *p* values were calculated using a linear mixed effects model, except for the Vehicle-ACY1083 comparison in ***E***, which did not pass a normality test and thus underwent a nonparametric Mann–Whitney test.

A model akin to the tau inclusion assay has been described wherein neuronal αSyn inclusions with similarity to those found in PD and other α-synucleinopathies form after seeding of mouse hippocampal neurons with recombinant αSyn PFFs. Mouse αSyn-positive and phospho-S129 αSyn-positive inclusions are readily detected 2 weeks after seeding of the neuronal cultures (Volpicelli-Daley et al., 2011; Volpicelli-Daley et al., 2014). We have optimized this neuronal assay to allow for compound assessment in a 384-well plate format and utilized this model to examine whether the HDAC6 inhibitors that reduced neuronal tau pathology might also affect αSyn inclusions. HDAC6 inhibitors and aSyn PFFs were added at DIV7, followed by an analysis of pathology at DIV22. As summarized in Figure 3 (see also Supplemental Materials, Fig. S2), staining of αSyn deposits that remained after detergent extraction of the cultures using antibodies that recognize total αSyn (9027) or phospho-Ser129 αSyn (81A) revealed a concentration-dependent inhibition that was separated from measures of neurotoxicity. The reduction of insoluble αSyn was further confirmed by analysis of insoluble protein fractions from the seeded neuron cultures that were treated in the absence or presence of ACY-1083 or ACY-775. In keeping with the immunocytochemical results, these compounds led to a significant ∼60% reduction of insoluble αSyn (Fig. 4*C*,*D*; *p*=<0.001 for ACY-775 and ACY-1083). Similarly, a significant reduction of phospho-S129 αSyn was observed with ACY-775 and ACY-1083 (*p* = 0.001 and 0.0022, respectively; Fig. 4*C*,*E*). HDAC6 inhibitor treatment also appeared to cause some increase in soluble αSyn, with ACY-1083 treatment reaching significance (*p* = 0.008; Fig. 4*A*,*B*). This increase in soluble αSyn may result from a decreased rate of recruitment of cytosolic αSyn into insoluble inclusions in the presence of the HDAC6 inhibitors.

**Figure 5.**
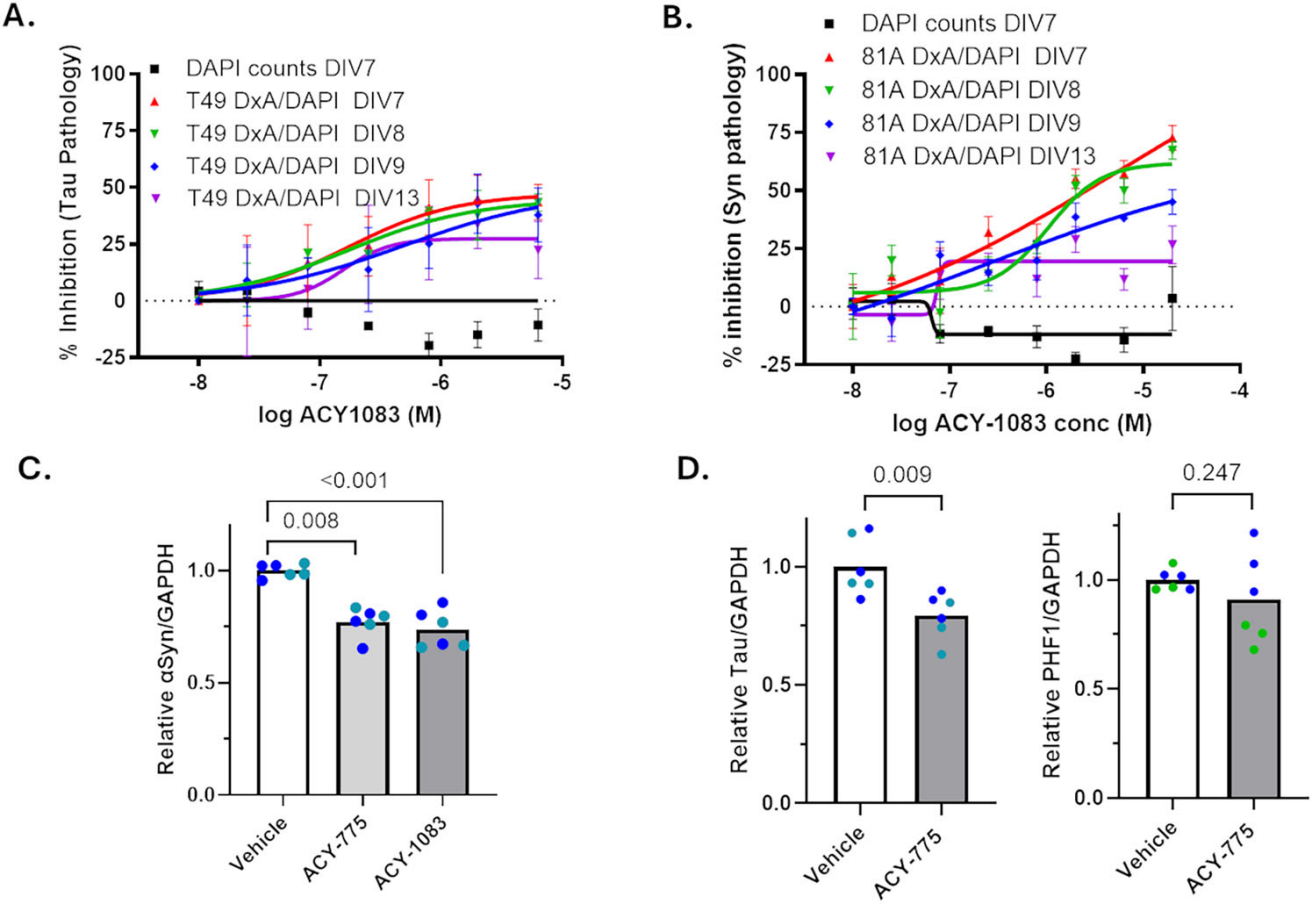
HDAC6 inhibitors reduced neuronal pathology formation when added days after initial AD-tau or αSyn PFF seeding, perhaps through a reduction of soluble neuronal tau and αSyn. ***A***, Multiple concentrations of ACY-1083 were added to rat cortical neuron cultures in triplicate wells at the time of AD-tau addition (DIV7), or 1 (DIV8), 2 (DIV9), or 6 d (DIV13) after AD-tau seeding. At DIV22, the cultures underwent assessment of T49-positive tau inclusions. In addition, DAPI-positive cell counts were determined in the cultures. ***B***, Multiple concentrations of ACY-1083 were added to mouse hippocampal neurons cultures in triplicate wells at the time of αSyn addition (DIV7), or 1 (DIV8), 2 (DIV9), or 6 d (DIV13) after αSyn PFF seeding. At DIV22, the cultures were assessed for 81A-positive phosphorylated αSyn inclusions. In addition, DAPI-positive cell counts were determined in the cultures treated with ACY-1083 at DIV7. Data were fit to a variable slope sigmoidal curve, with error bars representing standard error of the mean. ***C***, Mouse hippocampal neuron cultures in 12-well plates (two independent preparations) were treated with vehicle, or 6.7 µM of ACY-775 or ACY-1083, at DIV7 without seeding and cultured until DIV22. Lysates from identically treated duplicate wells in the same 12-well plate were pooled together as one sample, with three such pooled samples per treatment to yield triplicate samples. Equal protein amounts of the soluble fraction from the culture homogenates were then assessed by immunoblotting using the D37A6 (total mouse αSyn) antibody and a GAPDH antibody (see Supplemental Materials Fig. S3*A*), with results shown as relative αSyn/GAPDH. Each neuron batch is represented by a different color and each biological replicate by a separate data point. ***D***, Rat cortical neuron cultures in 12-well plates (two independent preparations) were treated with vehicle or 6.7 µM of ACY-775 at DIV7 without seeding and cultured until DIV22. Lysates from identically treated duplicate wells in the same 12-well plate were pooled together as one sample, with three such pooled samples per treatment to yield triplicate samples. Equal total protein amounts of the soluble fraction from the culture homogenates were then analyzed by immunoblotting using the T49 antibody (total rat tau) or the PHF-1 antibody (phospho-tau), as well as a GAPDH antibody (see Supplemental Materials Figs. S3*B,C*). Results are shown as relative αSyn/GAPDH or PHF1/GAPDH. Each neuron batch is represented by a different color and each biological replicate by a separate data point. *p* values in ***C*** and ***D*** were determined using a linear mixed effect model.

Conceptually, there are multiple potential mechanisms by which HDAC6 inhibition could reduce the formation or clearance of neuronal tau and αSyn inclusions, ranging from effects on initial fibril uptake and seeding to changes in fibrillization or degradation of misfolded species. To explore how HDAC6 inhibition might affect the early stages of inclusion formation, studies were conducted in which ACY-1083 was added to rat cortical neuron cultures at the time of AD-tau addition (DIV7) and up to 6 d after seeding with AD-tau (DIV13). As shown in Figure 5*A*, there was little difference in the concentration-dependent inhibition of neuronal tau pathology at DIV22 upon addition of ACY-1083 at the different times to the cultures, with reduced but still evident inhibition of tau inclusions when compound was added at DIV13. These data suggest that the compound inhibition of tau pathology was not due to effects on AD-tau uptake or blocking of initial seeding. A comparable study design was undertaken in αSyn PFF-seeded mouse hippocampal neurons, and similar results to those in the tau inclusion assay were observed. Addition of ACY-1083 at times after αSyn PFF seeding still caused appreciable reduction of phosphorylated αSyn inclusions, albeit with a greatly reduced effect when compound was added at DIV13 (Fig. 5*B*). These data suggest that HDAC6 inhibition lowers both tau and αSyn neuronal pathology through effects that occur after seed internalization.

The literature suggests that HDAC6 inhibition may lead to enhanced proteasomal clearance of certain tau species (Cook et al., 2012), as well as a reduction of aggregate-prone tau species due to enhanced tau acetylation that acts to inhibit tau phosphorylation (Cook et al., 2014). As the neuronal assays of tau and αSyn aggregation utilized here depend on lengthy (15 d) incubation periods to develop robust pathology, we cannot determine whether typical proteasome or autophagy inhibitors affect inclusion formation due to their toxic effects on neurons upon prolonged exposure. Moreover, HDAC6 inhibitor effects on soluble tau and αSyn species could be confounded by the concurrent recruitment of soluble species into seeded aggregates. Finally, there may be compensatory homeostatic changes in tau and αSyn expression that result from recruitment of the proteins into inclusions that could affect interpretation of HDAC6-mediated effects. To try to minimize such complications, the effects of HDAC6 inhibitor treatment on αSyn and tau were examined in nonseeded neuron cultures without inclusions using the same dosing paradigm as the seeded models (inhibitor or vehicle added at DIV7 with cells harvested at DIV22). Treatment of the nonseeded mouse hippocampal neurons with ACY-775 or ACY-1083 led to a ∼25% decrease of total αSyn (Fig. 5*C*, Supplemental Materials, Fig. S3*A*; *p* = 0.008 for ACY-775 and *p* < 0.001 for ACY-1083). Similarly, treatment of nonseeded rat cortical neurons with ACY-775 resulted in a comparable decrement in total tau (Fig. 5*D*, Supplemental Materials, Fig. S3*B*; *p* = 0.009.) An assessment of PHF1-positive phospho-tau (pS396/pS404) in the nonseeded neurons revealed a nonsignificant trend toward reduction upon ACY-775 treatment relative to vehicle-treated cultures (Fig. 5*D*, Supplemental Materials, Fig. S3*C*), although there was no evidence of preferential lowering of PHF1-tau relative to total tau. As most neuronal tau and αSyn are likely bound to their normal biological targets (i.e., microtubules for tau and synapse-related membranes for αSyn), a ∼25% lowering of the total amount of these proteins upon HDAC6 inhibitor treatment may mean larger reductions in the nonbound fractions of these proteins that could serve to elongate internalized seeds. In this regard, a reduction of total soluble αSyn or tau was not observed in seeded neurons upon HDAC6 inhibitor treatment, with soluble αSyn and tau being unchanged or even somewhat higher after HDAC6 inhibition (Figs. 2, 4). This may relate to differential rates of monomer recruitment into fibrils in the absence or presence of HDAC6 inhibitor, whereby a HDAC6 inhibitor-mediated decrease in tau and αSyn results in slower recruitment of monomers into elongating fibrils. This could explain the large reduction in inclusion burden observed in HDAC6 inhibitor-treated neurons, with less depletion of monomers over time than seen in vehicle-treated cultures (depicted schematically in Supplemental Materials, Fig. S3*D*). Although a reduction of soluble tau and αSyn may provide a possible explanation for the attenuated inclusion formation upon HDAC6 inhibition in seeded neurons, additional or alternative HDAC6 mechanisms are also possible.

### HDAC6 knockdown lowers tau and α-synuclein inclusions in primary neuron culture models

The observation that multiple HDAC6-selective inhibitors caused reductions in neuronal tau and αSyn inclusions implied that the compounds acted through an on-target mechanism. To further confirm this, studies were undertaken in which the neuronal culture models were treated with AAV1 harboring shRNA directed to HDAC6. Three commercially available AAV1 were obtained that contain 21-nucleotide complementary sequences to distinct regions of mouse HDAC6 mRNA (see Supplemental Materials, Table S1; referred to as AAV1-A, AAV1-B, and AAV1-C). In addition, AAV1 harboring a scrambled shRNA (AAV1-scr) was used as a negative control. Initial studies were conducted in mouse hippocampal neuron cultures at varying AAV1 concentrations to determine the amount of virus that could be added at DIV7 without evidence of neurotoxicity at DIV22, as assessed by light microscopy visualization of cultures. Based on these preliminary studies, all AAV1 samples showed evidence of toxicity when added at >6.25 × 10^7^ viral particles/ml, resulting in reduced cell density and disrupted neuronal processes. AAV1-C continued to show evidence of toxicity in the mouse neuron cultures at lower AAV1 concentrations, whereas AAV1-scr, AAV1-A, and AAV1-B were consistently free of evident toxicity in the mouse cultures at an AAV1 concentration of 3.12 × 10^7^/ml. To further evaluate the effects of AAV1-A and AAV1-B, additional immunocytochemistry studies were conducted with αSyn PFF-treated mouse hippocampal neuron cultures in which 3.12 × 10^7^ viral particles/ml were added at DIV6, followed by immunocytochemical staining at DIV22 to assess neuronal dendrites (MAP2 antibody) and astrocyte density (GFAP antibody). The latter were evaluated since the cultures contain a meaningful astrocyte population in addition to neurons by DIV22. As depicted in Supplemental Material, Figure S4*A*, neither AAV1-A nor AAV1-B had a detrimental effect on dendritic morphology (MAP2) relative to vehicle-treated cultures at this virus level. However, AAV1-B surprisingly caused a reduction of GFAP-positive astrocytes in the hippocampal neuron cultures. Neuron health can be negatively affected in cultures without astrocyte support, and astrocytes may play a role in the catabolism of fibrils added to the culture (Martini-Stoica et al., 2018; Filippini et al., 2021), such that a greater fibril burden may persist in cultures with reduced astrocytic content. Due to the potential confounds in the interpretation of results from studies with AAV1-B, we focused on examining the effects of AAV1-A and AAV1-scr on HDAC6 protein expression and αSyn pathology in the mouse neuron cultures.

**Figure 6.**
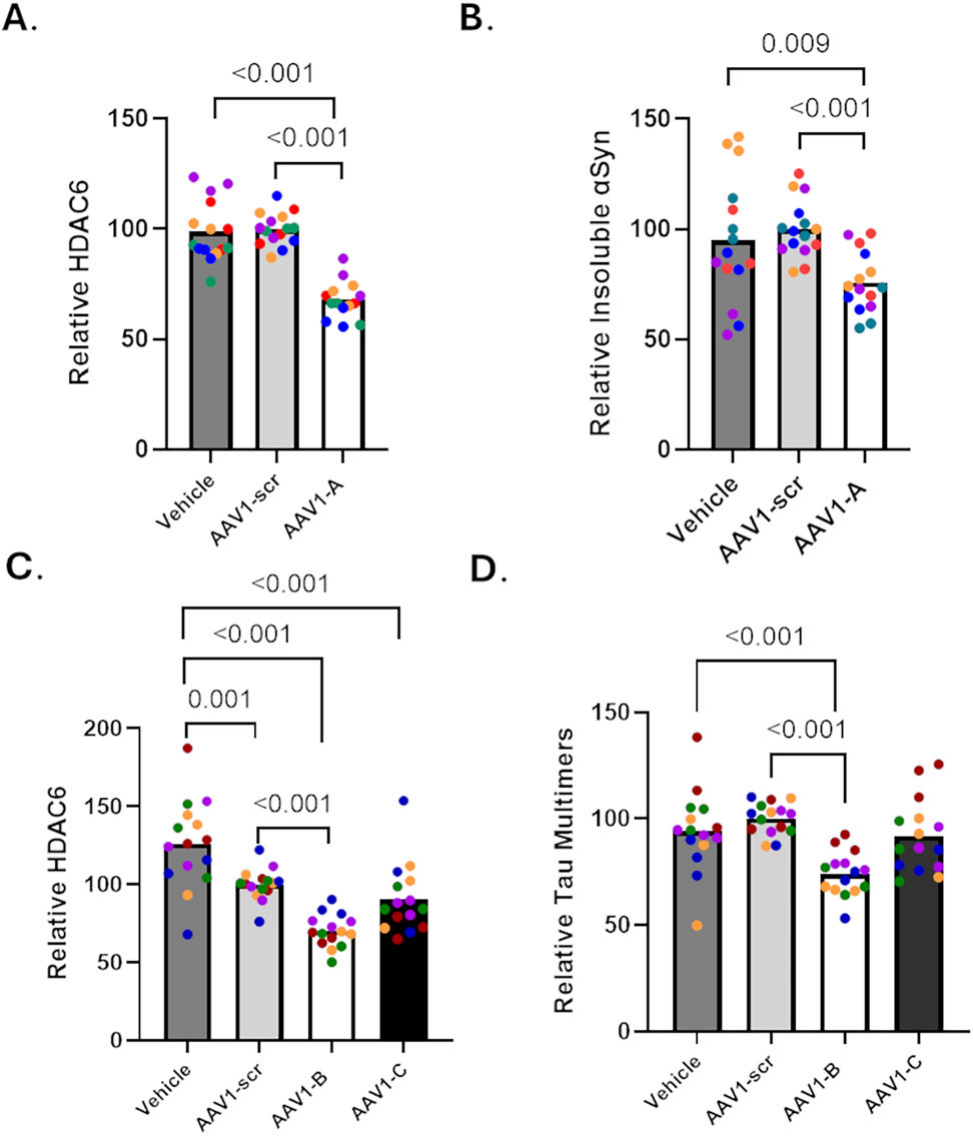
Reduction of HDAC6 protein expression led to attenuated neuronal αSyn and tau inclusions. Preparations of mouse hippocampal neurons were treated at DIV6 with AAV1 expressing shRNA directed to mouse HDAC6 (AAV1-A), AAV1 expressing scrambled shRNA (AAV1-scr), or no AAV1 (vehicle) followed by fibril treatment on DIV7 (5 separate neuron preparations with three independent biological replicates per treatment per neuron preparation). At DIV22, the culture wells were harvested and separated into RIPA-soluble and RIPA-insoluble fractions. Equal total protein amounts from the RIPA-soluble samples were run on SDS-PAGE followed by immunoblotting using HDAC6 and actin antibodies (Supplemental Data, Fig. S5*A*). Prior to immunostaining, the blots were probed with Ponceau S stain to visualize total protein in each lane (Supplemental Data, Fig. S5*B*). The integrated HDAC6 signal in each lane was divided by the total protein signal from Ponceau S staining of that lane. These values were then normalized to the mean of the AAV1-scr treatment group for comparison of treatment conditions. The results of AAV1 treatment on HDAC6 expression are shown in ***A***, with each neuron preparation depicted as a different color. The amount of αSyn in RIPA-insoluble fractions was determined using a mouse αSyn ELISA, and these values were normalized to the mean of the AAV1-scr treatment group. The results of AAV1 treatment on insoluble αSyn are shown in ***B***, with each independent study a different color. In addition, preparations of rat cortical neurons were treated at DIV6 with AAV1 expressing shRNA directed to mouse HDAC6 (AAV1-B and AAV1-C), AAV1-scr, or no AAV1 (vehicle) followed by fibril treatment on DIV7 (five separate neuron preparations with three independent biological replicates per treatment per neuron preparation). At DIV22, the culture wells were harvested and homogenized in RIPA. Equal total protein amounts from the RIPA homogenate samples were run on SDS-PAGE followed by immunoblotting using HDAC6 and actin antibodies (Supplemental Data, Fig. S5*C*). Prior to immunostaining, the blots were probed with Ponceau S stain to visualize total protein in each lane (Supplemental Data, Fig. S5*D*). Quantification of HDAC6 levels were as described above, and the results of AAV1 treatment on HDAC6 expression are shown in ***C***, with each neuron preparation a different color. The amount of multimeric tau (dimers and larger) in the homogenate samples was determined using a rodent tau multimer ELISA. These values were normalized to the mean of the AAV1-scr treatment group, with the results of AAV1 treatment on tau multimers shown in ***D***, with each neuron preparation a different color. All *p* values were calculated using a linear mixed effects model.

Multiple independent studies were conducted in the mouse hippocampal neuron model of induced αSyn pathology in which the cultures were treated at DIV6 in triplicate with 3.12 × 10^7^ viral particles/ml of AAV1-A or AAV1-scr, or vehicle only, followed by seeding with αSyn PFFs at DIV7. At DIV22, culture homogenates were prepared and separated into RIPA-soluble and RIPA-insoluble fractions, followed by assessment of HDAC6 protein by immunoblotting (Supplemental Materials, Fig. S5*A*) and insoluble mouse αSyn protein levels by ELISA. As summarized in Figure 6*A*, these studies revealed that AAV1-A treatment resulted in ∼30% mean reduction of HDAC6 protein levels relative to AAV1-scr or vehicle treatment (*p* < 0.001 for both). Notably, AAV1-A led to a significant ∼25% mean reduction of insoluble mouse αSyn relative to cultures treated with AAV1-scr or vehicle (Fig. 6*B*; AAV1-A vs Vehicle, *p* = 0.009; AAV1-A vs AAV1-scr, *p* < 0.001). It is not surprising that the overall reduction of insoluble αSyn in the cultures expressing HDAC6 shRNA was less than that observed upon treatment with HDAC6 small molecule inhibitors, since the highest concentrations of the HDAC6 inhibitors used in the neuronal assays likely fully inhibited HDAC6 activity whereas there was only a partial reduction of HDAC6 protein levels in the AAV1-A shRNA-expressing cultures. In aggregate, the results with multiple small molecule HDAC6 inhibitors and HDAC6 shRNA suggest that a reduction of HDAC6 activity results in attenuated neuronal αSyn pathology.

Of the purchased AAV1 expressing shRNA directed to mouse HDAC6, AAV1-C shRNA had a complete sequence match with rat HDAC6, whereas AAV1-B shRNA had a single nucleotide mismatch to the rat HDAC6 sequence. Finally, AAV1-A shRNA had a two-nucleotide mismatch to rat HDAC6. Because there is evidence that a one base pair mismatch in siRNA can still effectively reduce mRNA expression (Amarzguioui et al., 2003; Du et al., 2005), both AAV1-C and AAV1-B were examined for effect on HDAC6 levels and tau inclusions in the AD-tau-seeded rat cortical neuron cultures. Initial studies suggested that rat cortical neuron cultures could tolerate higher AAV1 exposures than the mouse hippocampal neurons, with up to 6.25 × 10^7^/ml viral particles/ml of AAV1-B and AAV1-C showing little evidence of neuronal damage (Supplemental Materials, Fig. S3*B*). Notably, treatment of the rat cortical cultures with AAV1-B at this concentration did not cause the dramatic loss of astrocytes observed with the mouse hippocampal cultures, nor did treatment with AAV1-C. Multiple independent studies were conducted with rat neuron cultures treated at DIV6 in triplicate with vehicle, or AAV1-B, AAV1-C, or AAV1-scr, at 6.25 × 10^7^/ml viral particles/ml, followed by addition of AD-tau at DIV7 and growth until DIV22. Total culture homogenates were then prepared, without separation into soluble and insoluble fractions, to allow for assessment of combined oligomeric and fibrillar tau species using a previously described tau multimer ELISA (Crowe et al., 2020; Gibbons et al., 2023). The culture homogenates were also analyzed for HDAC6 levels by immunoblotting. The scrambled shRNA was found to decrease HDAC6 protein by ∼20% relative to cultures not receiving AAV1 (*p* = 0.001; Fig. 6*C*, Supplemental Materials, Fig. S5*C*), presumably through a nonspecific effect that did not appear to affect overall cellular health since neuronal and astrocyte morphology appeared unaltered. Importantly, there was a 30% mean reduction of HDAC6 protein in cultures that received AAV1-B relative to cultures treated with AAV1-scr (*p* < 0.001). Somewhat surprisingly, AAV1-C was less effective at reducing HDAC6 levels in the rat neuron cultures, with a 10% lowering that was not significantly different than AAV1-scr. Notably, the reduction of HDAC6 expression with AAV1-B led to a significant 26% mean reduction in multimeric tau species in the rat neuron cultures relative to AAV1-scr (*p* < 0.001) and also a significant decrease in tau multimers/fibrils compared with vehicle treatment (*p* < 0.001; Fig. 6*D*). Consistent with diminished HDAC6 KD with AAV1-C, this shRNA did not cause a significant reduction of multimeric tau relative to vehicle or AAV1-scr.

Although only moderate KD of HDAC6 was observed in the mouse hippocampal and rat cortical neuron cultures treated with AAV1 expressing HDAC6 shRNA, there was good agreement between the extent of HDAC6 KD and the corresponding reduction of insoluble αSyn and multimeric tau species. These data further support the results obtained with the small molecule HDAC6 inhibitors, indicating that a reduction of HDAC6 activity results in an attenuation of both tau and αSyn pathological species.

### Assessment of HDAC6 inhibitor activity in the mouse brain

**Figure 7.**
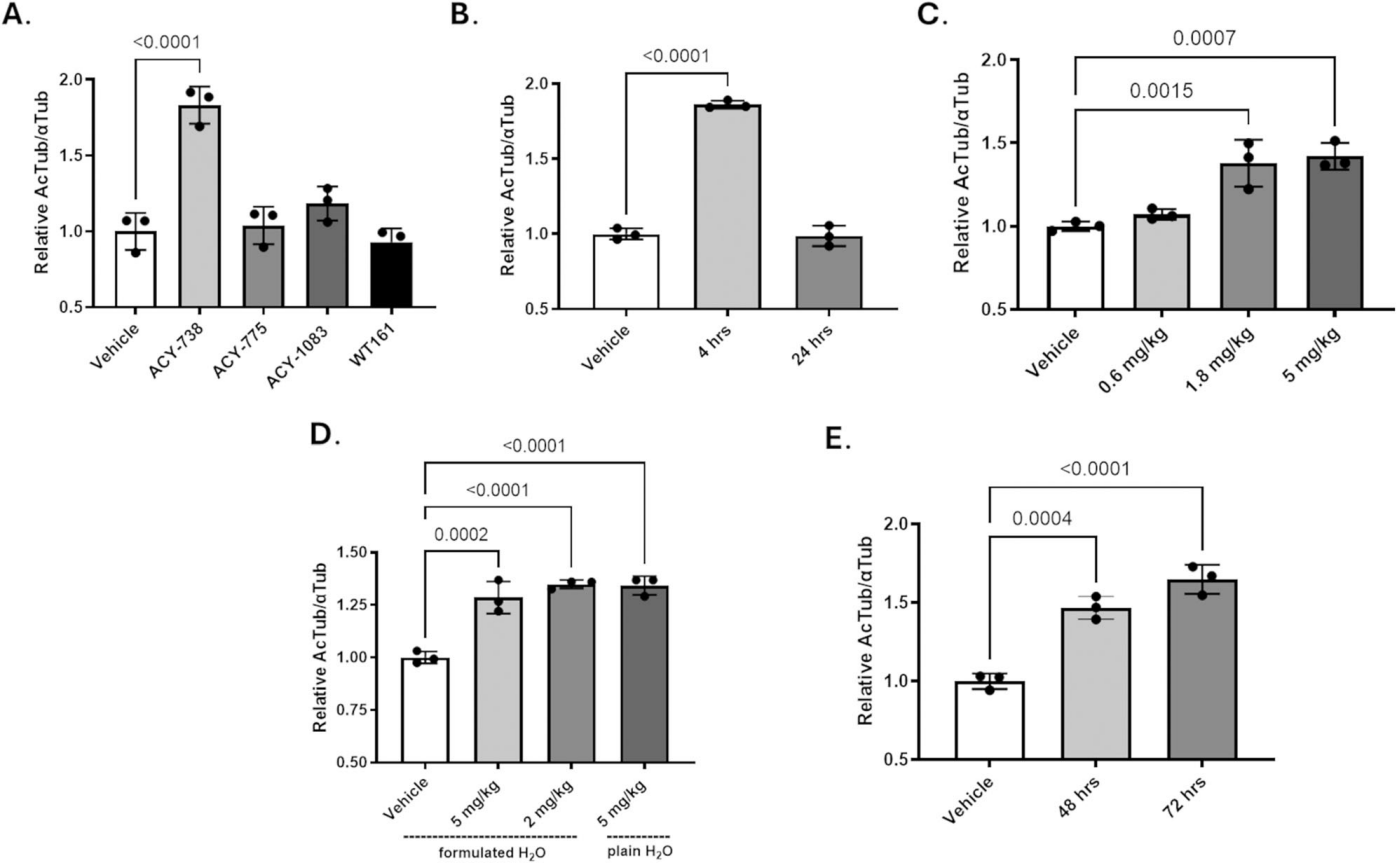
ACY-738 reduces brain HDAC6 activity when dosed orally. ***A***, HDAC6 inhibitors were administered via intraperitoneal injection to three WT mice for each compound at 25 mg/kg, with killing 4 h after dosing followed by preparation of brain homogenates. Brain AcTub and α-tubulin levels were assessed by ELISA and the AcTub/α-tubulin ratio expressed relative to mice that received vehicle only. ***B***, ACY-738 was administered by oral gavage to three WT mice at 25 mg/kg, and the brain AcTub/α-tubulin ratio was determined 4 and 24 h after dosing, with data expressed relative to mice that received vehicle treatment. ***C***, ACY-738 was administered by oral gavage to three WT mice at 0.6, 1.8, or 5 mg/kg, with brain AcTub/α-tubulin ratio determined 4 h after dosing. Data are expressed relative to mice that received vehicle treatment. ***D***, ACY-738 was provided to WT mice (*n* = 3) in formulated drinking water at a dose of 2 or 5 mg/kg (based on 5 ml of water consumption per day) or at 5 mg/kg in plain water. The brain AcTub/α-tubulin ratio was assessed 48 h after continued access to the ACY-738-containing water, with the data expressed relative to mice that received only compound-free formulated water. ***E***, WT mice (*n* = 3) were given continued access to 2 mg/kg of ACY-738 in drinking water for 48 or 72 h, followed by assessment of brain AcTub/α-tubulin ratio. The data are expressed relative to mice that received only compound-free water for 72 h. Error bars represent standard error of the mean. *p* values were calculated using a one-way ANOVA and a Dunnett's multiple-comparison test.

Existing commercially available HDAC6 inhibitors have generally been reported to have short half-lives in mice and in most cases relatively poor brain exposure. The literature suggested that ACY-738 (Jochems et al., 2014), ACY-775 (Jochems et al., 2014), ACY-1083 (McAlpin et al., 2022), and WT161 (Zhang et al., 2024) showed evidence of brain HDAC6 inhibitory activity. These compounds were assessed here for their ability to promote increased acetylation of brain α-tubulin within microtubules, one of the most abundant neuronal substrates of HDAC6 (LoPresti, 2020). In initial studies, each of these HDAC6 inhibitors or vehicle only were administered at 25 mg/kg via intraperitoneal injection into WT mice, followed by killing 4 h later with determination of acetylated-α-tubulin (AcTub) levels in brain homogenates. As shown in Figure 7*A*, a marked increase in brain AcTub was observed only in the mice that received ACY-738 (*p* < 0.0001), with a small but nonsignificant increase also seen with ACY-1083. ACY-738 is reported to bind HDAC6 with 1.7 nM affinity, with >100-fold selectivity over other class 1 HDACs (Jochems et al., 2014). However, the compound has a short plasma half-life of <0.5 h (Jochems et al., 2014). Thus, the increase in AcTub observed 4 h after administration may result from a persistence of HDAC6 inhibition. A possible explanation for this is that HDAC6-bound ACY-738 may have a slow off-rate that results in prolonged inhibition of HDAC6.

The testing of an HDAC6 inhibitor in mouse models of neurodegenerative disease would require multiple weeks of dosing, and thus oral compound administration in drinking water or chow would be a preferred route of administration. This is particularly true of a compound like ACY-738 that has a very short half-life, as dosing in food or water would result in frequent microdosing due to multiple feeding and drinking bouts each day. To test the oral bioavailability of ACY-738, WT mice received compound by oral gavage at 25 mg/kg. A significant increase in brain AcTub was seen 4 h after oral administration (Fig. 7*B*; *p* < 0.0001) that was comparable with that observed upon intraperitoneal dosing. The effect of a single oral dose of ACY-738 on tubulin acetylation was no longer apparent 24 h after administration (Fig. 7*B*), revealing that the compound-induced increase in AcTub, while longer than the compound half-life, is eventually lost over time. A subsequent study (data not shown) revealed that a single oral dose of 5 mg/kg ACY-738 increased brain AcTub to a level that was comparable with that observed at 25 mg/kg. To better define the lowest effective oral dose of ACY-738, the compound was given by oral gavage at 5, 1.8, and 0.6 mg/kg. Whereas the 1.8 and 5 mg/kg doses elicited comparable increases in brain AcTub 4 h after administration (*p* = 0.0015 and *p* = 0.0007, respectively), the 0.8 mg/kg dose failed to elevate brain AcTub (Fig. 7*C*). We subsequently investigated whether similar brain HDAC6 inhibition could be demonstrated upon administering the compound in drinking water. In initial studies, an additive mixture that has been previously employed to facilitate compound solubility and increase palatability (Duan et al., 2004) was added to the drinking water along with ACY-738. Administration via this formulated drinking water with a daily dose equivalent of 5 mg/kg (based on 5 ml of water consumption per mouse) or 2 mg/kg gave similar increases of brain AcTub after 48 h of access to water (Fig. 7*D*; *p* < 0.0001 and *p* = 0.0002, respectively). Moreover, a comparison of brain AcTub levels after treatment with 5 mg/kg of compound in plain water without additives revealed comparable inhibition to the formulated mix (Fig. 7*D*; *p* < 0.0001). Since the oral gavage studies indicated that a dose of ACY-738 below 2 mg/kg was ineffective (Fig. 7*C*), we selected 2 mg/kg as the minimal effective oral dose. A final study suggested that the ACY-738 was stable in water for at least 72 h, as mice that drank from a single 2 mg/kg preparation over this period had significantly increased brain AcTub (*p* < 0.0001) that was as comparable with that observed after 48 h exposure to the compound in water (*p* = 0.0004; Fig. 7*E*). These data indicated that sustained HDAC6 inhibition could be obtained by administration of ACY-738 in drinking water at a dose of 2 mg/kg, allowing for twice weekly changing of water preparations in longer term studies.

### Testing of ACY-738 in a WT mouse model of seeded tau and αSyn pathology

As noted, there are conflicting data on whether reduction of HDAC6 activity in the brain leads to a lowering of tau pathology in mouse models of disease. These prior studies typically utilized mouse models with overexpression of mutant tau, and there would be value in assessing HDAC6 inhibition in a mouse model of tau pathology with endogenous levels of tau expression, as seen in AD. In recent years, it has been shown that tau pathology can be initiated in WT mouse brain by stereotaxic injection of human brain-derived AD-tau, with subsequent formation of endogenous mouse tau pathology near the sites of injection and spreading to more distant brain regions over time (Guo et al., 2016; He et al., 2020). Moreover, αSyn pathology can also be induced in WT mice through intracerebral injection of mouse αSyn PFFs, with formation of intraneuronal αSyn inclusions near the injection site that spread temporally through neuronal networks (Luk et al., 2012b; Luk et al., 2012a; Nouraei et al., 2018). The effect of HDAC6 inhibition has not been extensively studied in mouse models of αSyn pathology, and thus there would be value in determining whether HDAC6 inhibition might reduce αSyn and/or tau pathology in seeded WT mice with endogenous levels of tau and αSyn expression. Accordingly, we induced tau and αSyn inclusions concurrently in WT mice through the intracerebral coinjection of AD-tau and αSyn PFFs (Luk et al., 2012b; Ardah et al., 2014; Bassil et al., 2021) and asked whether ACY-738 might reduce one or both of these neurodegenerative pathologies 3 months after brain seeding. Prior studies showed that tau and αSyn inclusions can be most readily observed near the injection sites at this time, with these pathologies spreading to more distant regions over longer time periods (Luk et al., 2012b; Ardah et al., 2014; Bassil et al., 2021). Importantly, the tau and αSyn inclusions that form in these mice not only model key pathologies of AD and PD but also mimic important aspects of known copathology in human disease. It has been estimated that ∼50% of AD patients also have αSyn pathology, and conversely a similar percentage of PD patients have tau copathology (Irwin and Hurtig, 2018; Robinson et al., 2018).

Two dosing paradigms were utilized in the WT mice that received injections of AD-tau and mouse αSyn PFFs into the hippocampus and overlying cortex. One group of mice (6 males and 6 females) comprised a preseeding treatment arm, as they were given access to drinking water containing ACY-738 (2 mg/kg equivalent dose) starting 1 d prior to brain injection of the tau and αSyn seeds. This group was then given ACY-738 through drinking water for 3 additional months after the brain injections. A second group of mice (5 males and 6 females) first received ACY-738 in drinking water (2 mg/kg) 1 week after the brain injection of AD-tau and αSyn PFFs, such that initial seeding of pathology by the injected material was already initiated. In fact, residual AD-tau and αSyn PFFs that are injected into the brain are largely cleared within 1 week (Luk et al., 2012a; Guo et al., 2016). This secondary prevention/intervention dosing group again received ACY-738 in drinking water for a total of 3 months after the brain injections. Finally, a vehicle group of AD-tau/αSyn PFF-injected mice (5 males and 6 females) received only compound-free water over the 3 month postinjection period. The body weights of all mice were evaluated weekly during the study period, and both the vehicle and compound-treated mice showed comparable body weight gain (Supplemental Materials, Fig. S6*A*).

Upon study completion, all mice were killed and perfused, with removal of brains for fixation and sectioning for evaluation of tau and αSyn pathology. In addition, organ weights from all animals were obtained at the study conclusion to determine whether there were overt morbidities resulting from the 3 months of ACY-738 dosing. Cognitive testing was not undertaken with the study mice, since there have been no reports of measurable learning and memory deficits in WT mice 3 months after intracerebral injection of AD-tau or αSyn PFFs, consistent with the absence of overt neuronal loss in the mice at this postinjection time (Guo et al., 2016; Nouraei et al., 2018). No differences were observed in any organ weights between the compound-treated groups and the vehicle only group (Supplemental Materials, Fig. S6*B*). The absence of any changes in overall body weight or organ weights indicated that ACY-738 was well tolerated by the mice.

Fixed sections (6 µm) spanning the brain were prepared from all study mice, and every 20 sections underwent immunohistochemical staining for αSyn pathology using the EP1536Y antibody that binds phosphorylated-S129 αSyn (Betemps et al., 2023) or for tau inclusions using the AT8 antibody that recognizes phosphorylated S202/T205 residues of pathologic tau (Zhang et al., 2012; Zhang et al., 2018). The majority of EP1536Y-positive αSyn pathology was found near the hippocampal injection site (Luk et al., 2012b), with additional sparse EP1536Y staining observed in other brain regions. Similarly, the most robust tau pathology was found within the ipsilateral hippocampus (Guo et al., 2016), with lesser ipsilateral cortical pathology and some AT8-positive staining in the contralateral hippocampus that was generally restricted to regions around bregma −3.64.

**Figure 8.**
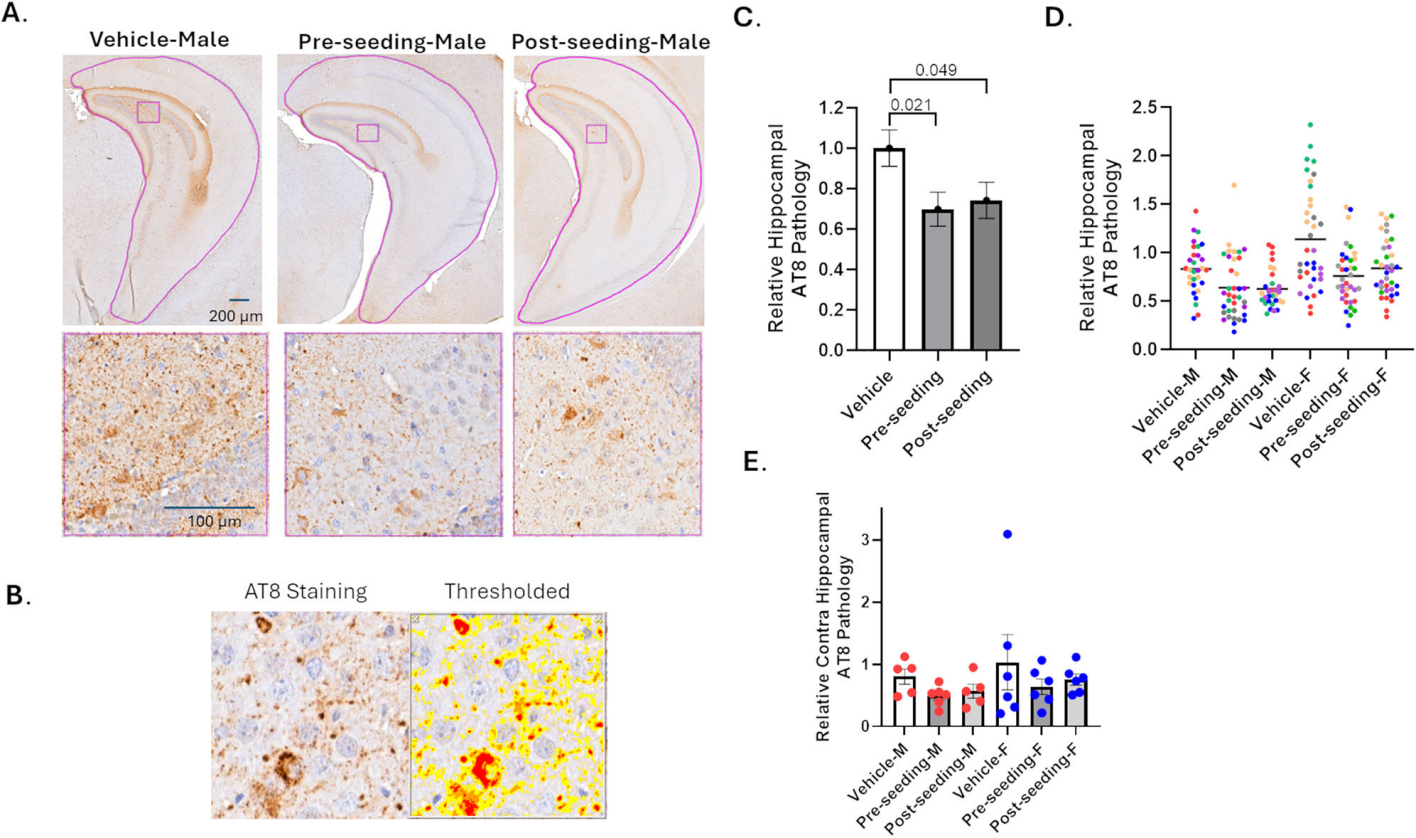
ACY-738 treatment reduced seeded tau pathology in the brains of WT mice. WT mice (males and females) received injections of a mixture of AD-tau and αSyn PFFs into the hippocampus and overlaying cortex. One group of mice (6 males and 6 females; preseeding dosing) were first provided access to ACY-738 (2 mg/kg equivalent) in drinking water 1 d prior to the intracerebral injections, whereas another group (5 males and 6 females; postseeding dosing) were first provided ACY-738 in drinking water 7 d after brain seeding. A third group of AD-tau/αSyn PFF-injected mice received only compound-free water (5 males and 6 females; vehicle dosing). All mice were killed 3 months after AD-tau/αSyn PFF injections, and brain sections were stained with AT8 antibody to visualize phospho-tau-positive pathology. ***A***, Images showing the annotated ipsilateral hippocampus at bregma −3.08 from representative male mice from each treatment group, with a higher magnification image of the boxed area shown to better visualize tau pathology. ***B***, Example image showing AT8-stained tau pathology and the threshold applied to remove background staining prior to quantification of the integrated signal within the colored regions. ***C***, The relative ipsilateral hippocampal AT8-positive tau pathology from each evaluated section was normalized to the mean of the vehicle-treated group at that bregma level, and the normalized value for each section from each mouse was utilized in a linear mixed effects model. The mean of each treatment group is graphed, with error bars representing the standard error of the mean. ***D***, The individual normalized data for each quantified brain section are plotted to show the separate male and female values. Each study mouse within a treatment group is shown with a different color, with multiple bregma levels evaluated per mouse. ***E***, The amount of AT8-positive tau pathology was assessed in the contralateral hippocampus at bregma −3.64, with the data from each mouse plotted by sex and treatment group. Error bars represent standard error of the mean.

For analysis of tau inclusions, sections from six different bregma levels spanning bregma −2.18 to −3.64 were quantified for AT8-positive staining from each study mouse. Quantification focused on the ipsilateral hippocampus, as this region not only contained the most robust pathology, but it could also be readily demarcated to allow for more precise comparisons between treatment groups. The ipsilateral hippocampus was manually annotated on each image (Fig. 8*A*, Supplemental Data, Fig. S7), and the amount of AT8-positive signal was quantified after image thresholding (Fig. 8*B*) by a researcher masked to treatment type. The resulting data from each bregma level (expressed as AT8-positive area × optical density divided by the annotated hippocampal area) was normalized to the mean of the vehicle-treated group at each bregma, thereby allowing brain sections from all bregma levels to be combined to assess potential group differences. Comparing the relative AT8-positive tau pathology (example images from each treatment group at bregma −3.08 shown in Fig. 8*A*) revealed that both preseeding (*p* = 0.021) and postseeding (*p* = 0.049) dosing with ACY-738 led to significant reductions of tau pathology when both sexes were combined (Fig. 8*C*). The mean reduction of AT8 staining was 30% in the preseeding group and 26% in the postseeding treatment group. Thus, the lowering of tau pathology was similar when ACY-738 was administered before or after intracerebral seeding, in keeping with the results obtained in the neuronal tau inclusion model where compound addition days after AD-tau seeding still led to reduced pathology. The individual measurements for each bregma are shown by sex in Figure 8*D*, with each mouse within a treatment group depicted by a different color. The female mice had greater overall ipsilateral hippocampal tau pathology than the male mice when all groups were compared (*p* = 0.028), but there was not a significant difference in the extent of compound inhibition between sexes. The reason(s) for the sex difference on the overall amount of tau pathology is unclear, although it has been suggested that female AD patients show greater tau pathology than males (Buckley et al., 2020; Xu et al., 2025). As noted, AT8-positive staining was also observed in the contralateral hippocampus in sections at bregma −3.64, and this pathology was quantified from each study mouse. Because differences in contralateral hippocampal pathology could not be averaged over multiple bregma levels, the analysis did not yield statistically significant differences between the treatment groups. However, the overall trends were comparable with those observed in the ipsilateral hippocampus (Fig. 8*E*).

**Figure 9.**
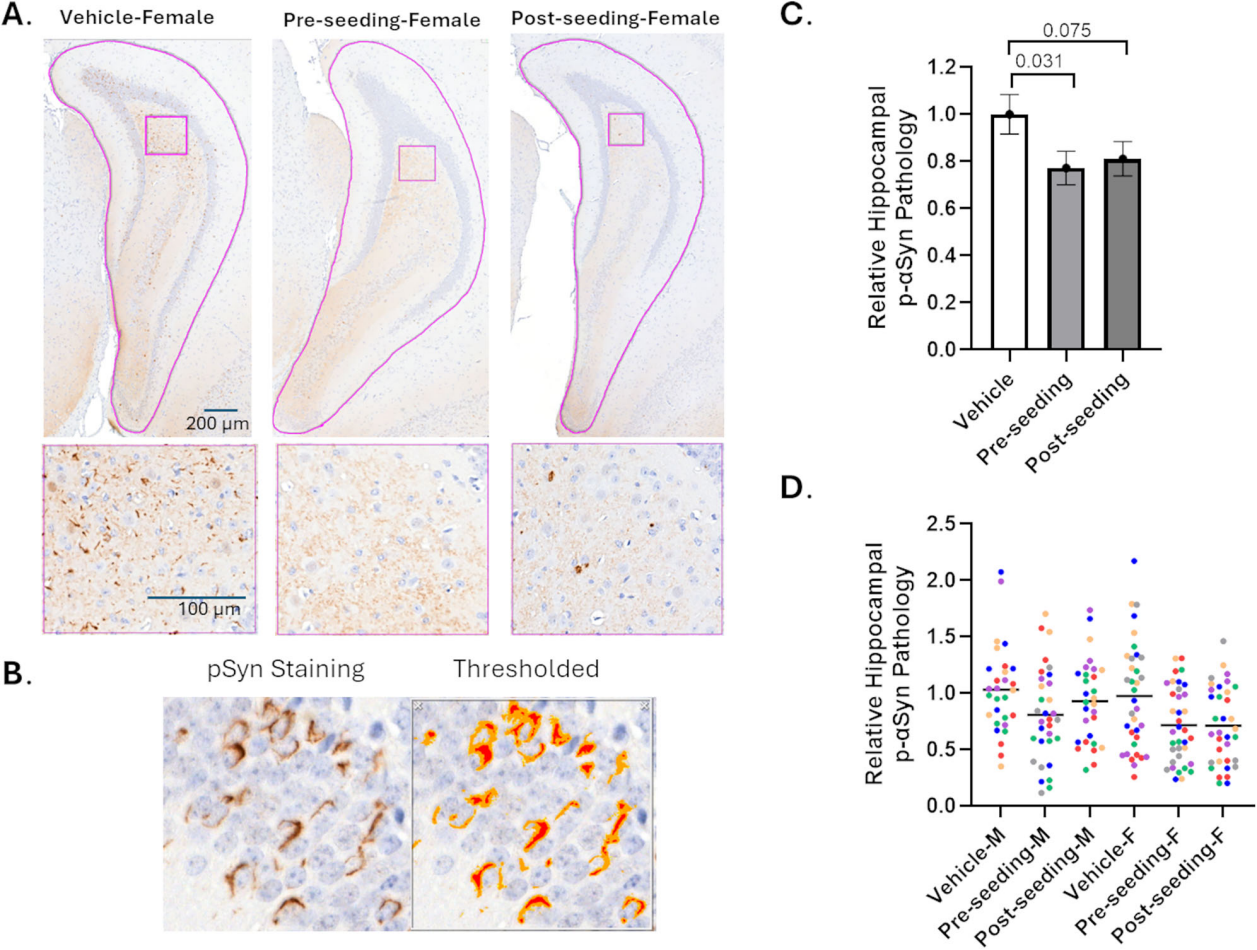
ACY-738 treatment reduced seeded αSyn pathology in the brains of WT mice. Brain sections from the mice described in the Figure 8 legend were stained with EP1536Y antibody to detect phosphorylated αSyn pathology. ***A***, Images showing the annotated ipsilateral hippocampus at bregma −3.80 from representative female mice from each treatment group, with a higher power image of the boxed area shown to better visualize αSyn pathology. ***B***, Example image showing EP1536Y-stained αSyn pathology and the threshold applied to remove background staining prior to quantification of the integrated signal within the colored regions. ***C***, The relative ipsilateral hippocampal EP1536Y-positive αSyn pathology from each evaluated section was normalized to the mean of the vehicle-treated group at that bregma level, and the normalized value for each section from each mouse was utilized in a linear mixed effects model. The mean of each treatment group is graphed, with error bars representing standard error of the mean. ***D***, The individual data for each quantified brain section are plotted to show the separate male and female values. Each study mouse within a treatment group is shown with a different color, with multiple evaluated bregma per mouse.

The assessment of αSyn pathology in the study mice using the EP1536Y antibody to detect phosphorylated αSyn was essentially as described above for quantification of tau pathology, with six bregma levels spanning −1.94 to −3.80 evaluated (Supplemental Material, Fig. S8). Again, the ipsilateral hippocampus from each bregma image was manually annotated (example images from bregma −3.80 in Fig. 9*A*) and the amount of αSyn pathology was measured after thresholding to eliminate background staining (Fig. 9*B*) by a researcher masked to treatment group. The integrated αSyn signal was normalized at each bregma to the vehicle treatment mean, and a comparison of treatment groups revealed a significant reduction of αSyn inclusions in the preseeding (*p* = 0.031) treatment group. There was also a strong trend toward attenuated αSyn pathology in the postseeding treatment group relative to the vehicle group (Fig. 9*C*; *p* = 0.075). The mean reduction of αSyn pathology upon ACY-738 treatment was again relatively similar between the preseeding and postseeding dosing schemes (23 and 19%, respectively), although of somewhat lesser magnitude than the compound-mediated lowering of tau pathology. A comparison of each evaluated brain section for male and female mice, with each mouse within a treatment group depicted by a different color (Fig. 9*D*), revealed no overall sex-dependent difference in total αSyn pathology. Likewise, there was not a significant difference in compound effect between the sexes, although there was a trend toward greater compound effect in female mice. We note that the percentage of hippocampal area that was positive for phosphorylated αSyn pathology was ∼10-fold less than that for tau pathology, thereby resulting in somewhat less robust αSyn measurements that may have somewhat reduced the sensitivity of the quantification. Nonetheless, the totality of the mouse efficacy data reveals that ACY-738 treatment led to moderate reductions of both tau and αSyn pathologies in the seeded WT mouse model.

## Discussion

There have been important advancements in the development of immunotherapeutics that reduce Aβ plaques and slow clinical progression in AD (Sims et al., 2023; van Dyck et al., 2023). However, monoclonal antibodies and other agents directed to tau have thus far failed to show benefit in AD and tauopathy clinical trials (Jabbari and Duff, 2021; Panza et al., 2023). Similarly, there are presently no approved drugs that slow disease progression in PD and monoclonal antibodies directed to αSyn species have been unsuccessful in clinical testing (Xiao and Tan, 2023).

As described here, we found that reducing HDAC6 activity can attenuate the amount of tau and αSyn pathology in primary neuron culture models. These in vitro models of tau and αSyn pathology depend on seeded formation of intraneuronal inclusions in WT neurons, and there is increasing evidence that seeded propagation of pathology through neuronal networks explains the stereotypical progression of disease observed in neurodegenerative tauopathies and α-synucleinopathies (Vaquer-Alicea and Diamond, 2019; Uemura et al., 2020). Thus, the neuron culture models utilized here may more authentically represent the process of tau and αSyn inclusion formation than alternative models that rely on the overexpression of mutated forms of these disease-associated proteins.

There have been prior reports of reduced HDAC6 activity attenuating tau species through increased chaperone-dependent proteolysis (Cook et al., 2012). Similarly, HDAC6 inhibition may affect tau fibrillization and turnover through increased tau acetylation although it is unclear whether this enhances or inhibits fibrillization (Min et al., 2010; Cohen et al., 2011; Cook et al., 2014; Min et al., 2015; Trzeciakiewicz et al., 2017). Our data suggests that HDAC6 inhibition may lower the formation of seeded pathology through a reduction of soluble tau and αSyn species that can elongate internalized seeds. However, it is possible that HDAC6 inhibition may affect the formation of αSyn and tau pathologies through additional or alternative means.

The consequences of reducing HDAC6 activity in tau transgenic mouse models are quite inconsistent. While there is evidence of small molecule HDAC6 inhibitors lowering mouse tau pathology (Choi et al., 2020; Onishi et al., 2021), an HDAC6 inhibitor was also reported to lower total tau without reducing silver-positive tau inclusions (Selenica et al., 2014). Moreover, reductions of HDAC6 expression have not resulted in reduced mouse tau pathology, as ∼70% lowering of HDAC6 through intracerebroventricular injection of anti-sense oligonucleotides in tau transgenic mice did not alter tau acetylation, phospho-tau levels, or tau aggregation (Valencia et al., 2021). Furthermore, crossing HDAC6 knock-out mice with tau transgenic mice led to increased tau pathology and shortened lifespan, and the authors proposed an HDAC6-dependent mechanism that reduces toxic tau species (Trzeciakiewicz et al., 2020). It is unclear how to reconcile these disparate observations of HDAC6 modulation on tau pathology in transgenic mouse models. Perhaps >70% reduction of brain HDAC6 is required to observe a meaningful lowering of tau inclusions in adult tau transgenic mice. It is also conceivable that HDAC6 knock-out in mice overexpressing mutant tau leads to developmental changes that exacerbate tau brain pathology in ways not observed when HDAC6 is inhibited in adulthood. In this regard, a divergence of outcomes has been observed upon HDAC6 knock-out and small molecule HDAC6 inhibition in other model systems (Concors et al., 2024).

There have been few studies investigating the effect of HDAC6 inhibition on αSyn pathology. There is a report (Francelle et al., 2020) of an HDAC6 inhibitor causing reduced phospho-αSyn and total αSyn levels after substantia nigra injection with AAV expressing αSyn, although it is unclear whether αSyn aggregates were observed in this model. Our findings that reduced HDAC6 activity attenuated both αSyn and tau inclusions in neuronal models, and the conflicting literature on HDAC6 regulation of tau pathology, led us to further investigate the consequences of HDAC6 inhibition in a WT mouse model with seeded tau and αSyn inclusions. To identify a suitable HDAC6 inhibitor for efficacy testing, several HDAC6 inhibitors with reported brain activity were tested to determine whether they could enhance brain AcTub levels (LoPresti, 2020). Of these, only ACY-738 led to a significant increase in brain AcTub at 25 mg/kg. Additional characterization of ACY-738 revealed that oral administration in drinking water at a daily 2 mg/kg dose resulted in elevated brain AcTub, thus providing a brain-active HDAC6 inhibitor that could be administered for prolonged dosing periods.

The ability of ACY-738 to affect tau and/or αSyn inclusions in vivo was assessed in a model in which both of these pathologies were induced concurrently through the intracerebral injection of AD-tau and αSyn PFFs into WT mice (Luk et al., 2012b; Guo et al., 2016; Bassil et al., 2021). Two different dosing schemes were utilized, with ACY-738 administered via drinking water starting either 1 d prior to or 1 week after the brain injections. The former dosing scheme modeled a preventative treatment regimen. In contrast, since brain-injected AD-tau and αSyn PFFs are essentially cleared within 1 week (Luk et al., 2012a; Guo et al., 2016), the initiation of dosing 1 week after seed injection should not affect initial seeding of brain cells and thus can be viewed as a secondary prevention/interventional treatment. In both dosing groups, mice were given water containing ACY-738 for 3 months after the intracerebral seeding of tau and αSyn pathologies (or plain water in the control group). Immunohistochemical evaluations revealed that the greatest AT8-positive phospho-tau and EP1536Y-positive phospho-αSyn staining were found, as expected, in the ipsilateral hippocampus near the area of coinjected AD-tau and αSyn PFFs. To allow for a robust analysis, quantification of these pathologies was confined to the ipsilateral hippocampus due to the abundant staining and the ability to readily annotate this region to allow comparable mouse-to-mouse evaluations. Notably, a statistically significant reduction of tau inclusions was observed with both pre- and postseeding ACY-738 treatment, whereas αSyn inclusions were significantly reduced by AC-738 in the preseeding group, with a strong trend toward reduction after postseeding treatment. These results generally agree with those from the neuron culture models, where tau and αSyn inclusions were reduced even when HDAC6 inhibitor was added days after initial seeding. These observations suggest that HDAC6 inhibitors do not affect the initial seed uptake and instead may interfere with later postseeding elongation, perhaps by promoting the clearance of soluble tau and αSyn species.

The reduction of tau and αSyn pathologies in mice dosed with ACY-738 was significant but moderate, with ∼30% reduction of tau pathology and somewhat lesser reduction of αSyn inclusions. This is less than the 50–70% inhibition of these pathologies observed upon HDAC6 inhibitor treatment of the neuronal culture models. The WT mouse model of seeded pathology, while not dependent on overexpression of mutant tau or αSyn, may nonetheless represent a challenging test for pathology-modifying treatments. The mice received a bolus brain injection containing µg quantities of AD-tau and αSyn PFFs, initiating robust local seeding of endogenous mouse tau or αSyn. In human disease, such high local concentrations of pathological seeds would seem unlikely, and the spread of pathology occurs over longer timespans. Moreover, ACY-738 has a <30 min half-life in mice and compound administration in drinking water almost certainly resulted in periods of low HDAC6 inhibition, as mice consume much greater amounts of food and water in their night phase (Jensen et al., 2013). Thus, a brain-penetrant HDAC6 inhibitor with improved metabolic stability that could provide consistent HDAC6 inhibition might lead to even greater reductions of tau and αSyn deposits.

The ability to reduce both tau and αSyn pathologies with a single small molecule HDAC6 inhibitor has therapeutic appeal, not only because such a molecule might be used to treat both tauopathies and α-synucleinopathies, but also because these pathologies often coexist in patients. It has been estimated that ∼50% of AD patients have αSyn pathology and conversely a similar percentage of PD patients have tau copathology (Irwin and Hurtig, 2018; Robinson et al., 2018). These copathologies are likely to contribute to the disease course and having a drug that could inhibit both tau and αSyn inclusions might provide additional benefit beyond a drug that only targets the primary disease pathology. The potential benefits of a therapeutic approach must be considered in the context of patient safety, and in this regard HDAC6 knock-out mice develop normally, with minor effects on bone density (Zhang et al., 2008) and reported reductions in anxiety and depressive behavior (Fukada et al., 2012). Moreover, HDAC6-selective drug candidates appear to be reasonably well tolerated in human cancer patients (Yee et al., 2016; Tsimberidou et al., 2021). We observed no adverse effects after 3 months of dosing with ACY-738 in mice, with normal body and organ weights and no gross behavioral changes. Thus, it appears that prolonged HDAC6 inhibition might be safely tolerated and HDAC6 inhibitors may have potential for the treatment of neurodegenerative tauopathies and α-synucleinopathies. Further testing of brain-penetrant HDAC6 inhibitors, particularly those with improved metabolic stability, could provide additional validation of this therapeutic approach for AD, PD, and related neurodegenerative diseases.
